# ﻿Culturable fungi from urban soils in China II, with the description of 18 novel species in Ascomycota (Dothideomycetes, Eurotiomycetes, Leotiomycetes and Sordariomycetes)

**DOI:** 10.3897/mycokeys.98.102816

**Published:** 2023-06-29

**Authors:** Zhi-Yuan Zhang, Xin Li, Wan-Hao Chen, Jian-Dong Liang, Yan-Feng Han

**Affiliations:** 1 Institute of Fungus Resources, College of Life Sciences, Guizhou University, Guiyang 550025, China Guizhou University Guiyang China; 2 College of Eco-Environmental Engineering, Guizhou Minzu University, Guiyang 550025, China Guizhou Minzu University Guiyang China; 3 Basic Medical School, Guizhou University of Traditional Chinese Medicine, Guiyang 550025, China Guizhou University of Traditional Chinese Medicine Guiyang China

**Keywords:** Fungal taxonomy, keratinophilic fungi, morphological characters, phylogeny, 18 new taxa

## Abstract

As China’s urbanisation continues to advance, more people are choosing to live in cities. However, this trend has a significant impact on the natural ecosystem. For instance, the accumulation of keratin-rich substrates in urban habitats has led to an increase in keratinophilic microbes. Despite this, there is still a limited amount of research on the prevalence of keratinophilic fungi in urban areas. Fortunately, our group has conducted in-depth investigations into this topic since 2015. Through our research, we have discovered a significant amount of keratinophilic fungi in soil samples collected from various urban areas in China. In this study, we have identified and characterised 18 new species through the integration of morphological and phylogenetic analyses. These findings reveal the presence of numerous unexplored fungal taxa in urban habitats, emphasising the need for further taxonomic research in urban China.

## ﻿Introduction

Biodiversity has always been a hot area of research in ecology and biology. Fungi represent one of the most diverse groups of microorganisms on the planet, with an essential role in ecosystem processes and functioning (Hyde et al. 2020a). At the same time, fungi have a significant influence on human society. On the one hand, they are able to produce a large number of secondary metabolites that can be used by humans, such as various antibiotics and enzymes (Uchida et al. 2005; Hoffmeister and Keller 2007; El-Gendi et al. 2022; Mapook et al. 2022). They also infect humans, animals and plants, bringing great harm to human health and the national economy (Fisher et al. 2012; Fisher et al. 2020; Fones et al. 2020). To date, the total number of fungal species is still a prolonged debate. While numerous studies have explored fungal diversity in marine, cave, forest, volcanic, mountain, desert, freshwater aquatic systems, lakes, grasslands and indoor environments (Hyde et al. 2020a), their distribution in urban environments seems to have been overlooked.

Urbanisation is an inevitable trend in humanity’s development and is an important symbol of the progress made in science and technology (Wang et al. 2018). Urbanisation has swept across the globe over the last several decades (Berry 2008). The rate of urban expansion is currently at an unprecedented level and the migration of people from rural to urban regions leads to increased anthropogenic changes to the urban environment. These changes may include alterations in land use, the establishment of transportation networks and the management of urban soil and vegetation (Hoyt 1939; McDonnell and Pickett 1990; Antrop 2004). As urbanisation continues to expand worldwide (as noted by Rydin et al. 2012), urban soil fungi have become increasingly important in relation to human health and environmental concerns (Grimm et al. 2008). Due to the variety of urban soils, diverse habitats, rapid urbanisation and high population density, the study and investigation of the diversity of soil fungi in different cities in China will provide valuable scientific data for understanding their ecological functions and maintaining public health safety.

As the foundation of all fungal research, accurate identification and taxonomy for the fungal species are the primary and important task. Morphology is the traditional method for species classification. However, with the dramatic increase in species, it is very difficult to identify the fungal species from morphology alone. Recently, there has been an increase in the use of DNA barcoding or DNA classification methods to address the identification of specific taxa (Hebert et al. 2003; Tautz et al. 2003). ITS rDNA has been the common barcoding marker of fungal species (Schoch et al. 2012; Stielow et al. 2015; Vu et al. 2016). Polygenic phylogeny also has been used widely for species identification; for example, Wang et al. (2019) studied phylogenetic re-evaluation of *Thielavia* and proposed a new family and many new species. Hou et al. (2023) redisposition of acremonium-like fungi in Hypocreales combined morphological characterisation and multilocus phylogenetic analysis. Peng et al. (2023) reported eight new species of *Acrophialophora*, based on multilocus phylogenetic analysis. Therefore, the fungal taxonomy combining DNA-based approaches and morphological characterisation has been a widely accepted and used method (Orr et al. 2020).

In our investigation of keratinophilic fungi from urban soils in southern China, we have isolated and identified a large number of these fungi after using hair baiting enrichment treatment and reported several new taxa, for example, *Plectosphaerellaguizhouensis* and *P.nauculaspora* (Zhang et al. 2019a), *Gongronellasichuanensis* (Zhang et al. 2019b), *Ctenomycesalbus*, *C.obovatus* and *C.peltricolor* (Zhang et al. 2019c), *Geomycesobovatus*, *Pseudogymnoascussinensis* and *Solomycessinensis* etc. (Zhang et al. 2020b), *Cunninghamellaguizhouensis* (Zhang et al. 2020c), *Pseudogymnoascuscatenatus*, *Solomycesguizhouensis* and *Zongqiasinensis* etc. (Zhang et al. 2021b), *Arthrographismultiformispora* (Li et al. 2022a), *Keratinophytonchongqingense* and *K.sichuanense* (Li et al. 2022b), *Pseudogeomyceslindneri* and *Pseudogymnoascuscampensis* (Zhang et al. 2023a), *Paraneoaraneomycessinensis* and *Pochoniasinensis* (Zhang et al. 2023b). Following our previous studies, based on morphological characters and polygenic analysis, 18 new species were identified and described in this study. The new taxa belong to six orders (Eurotiales, Hypocreales, Onygenales, Thelebolales, Venturiales and Xylariales), eight families (Arthrodermataceae, Aspergillaceae, Bionectriaceae, Microdochiaceae, Nectriaceae, Niessliaceae, Sympoventuriaceae and Thelebolaceae) and 11 genera (*Aspergillus*, *Clonostachys*, *Cyanonectria*, *Echinocatena*, *Fusarium*, *Idriella*, *Nannizzia*, *Niesslia*, *Penicillium*, *Pseudogymnoascus* and *Talaromyces*) in the Pezizomycotina. This study helps in determining their ecological roles in the ecosystem and also contributes to the accumulation of new scientific resources for future research.

## ﻿Materials and methods

### ﻿Sample collection and fungal isolation

Soil samples were collected from the green belts of hospitals, parks and university campuses in some cities in southern China. Samples were collected from 3–10 cm below the soil surface, placed in Ziploc plastic bags, brought back to the laboratory and processed immediately. The soil samples were mixed with clean, sterile, chicken feathers, approximately 2 cm long and moistened with sterile water (Li et al. 2022b). The samples were then incubated in the dark at 23–27 °C for 1 month. For fungi isolation, 2 g of each of the collected samples was suspended in 20 ml of sterile water in a 50 ml sterile conical flask. The conical flasks were thoroughly shaken using a Vortex vibration meter. The suspension was then diluted to a concentration of 10^-4^. Then, 1 ml of the diluted sample was transferred to a sterile Petri dish and modified Sabouraud’s dextrose agar (SDA; peptone 10 g/l, dextrose 40 g/l, agar 20 g/l, 3.3 ml of 1% Bengal red aqueous solution) medium containing 50 mg/l penicillin and 50 mg/l streptomycin was added and mixed. The plates were incubated at 25 °C for 1–2 weeks and single colonies were selected from the plates and inoculated to new potato dextrose agar (PDA, potato 200 g/l, dextrose 20 g/l, agar 20 g/l) plates. The ITS regions of all isolates were sequenced and BLASTn searched in NCBI and assigned to a potential genus and species. According to Zhang et al. (2021a), the strains whose ITS sequences are less than 97% in the similarities to the closest strain were recognised as potential new species, which were further identified by combining morphological characterisation and phylogenetic analyses.

### ﻿Morphological study

Strains of potentially new species were transferred to new plates of potato dextrose agar (PDA, Bio-way, China), malt extract agar (MEA, Bio-way, China) and oatmeal agar (OA, Bio-way, China) and were incubated at 25 °C for examining their colony morphology and microscopic morphology. The colony diameters and morphologies were determined after 14 days and the colony colours on the surface and reverse of inoculated Petri dishes were assessed according to the Methuen Handbook of Colour (Kornerup and Wanscher 1978). The characterisation and measurement of fungal microscopic characteristics were performed in 25% lactic acid. Images were obtained using an optical microscope (OM; DM4 B, Leica, Germany) with differential interference contrast (DIC). Taxonomic descriptions and nomenclature were deposited in MycoBank (https://www.mycobank.org; accessed on 25 May 2022). Type collections of the novel species are deposited in the Mycological Herbarium of the Institute of Microbiology, Chinese Academy of Sciences, Beijing, China (HMAS; (https://nmdc.cn/fungarium/). The ex-type living cultures and other isolates are deposited in the China General Microbiological Culture Collection Center (CGMCC; https://www.cgmcc.net/english/; accessed on 7 April 2022) or at the Institute of Fungus Resources, Guizhou University (GZAC), Guiyang City, Guizhou, China.

### ﻿DNA extraction, PCR amplification and sequencing

Total genomic DNA was extracted from fungal mycelia using a BioTeke fungus genomic DNA extraction kit (DP2032, BioTeke, Beijing, China) following the manufacturer’s instructions. The internal transcribed spacers (ITS) are widely used in fungal biodiversity and phylogenetic studies (Schoch et al. 2012; Stielow et al. 2015; Vu et al. 2016) and combining ITS and LSU improves species discrimination (Heeger et al. 2018; Vu et al. 2019); thus, ITS and the 28S nrRNA locus (LSU) sequences of all isolates were sequenced. In addition, more loci are often needed for specific taxa to obtain higher accuracy, so this study also amplified different loci for different taxa, such as β-tubulin (*TUB*), RNA polymerase II subunit (RPB2) and Twenty S rRNA accumulation (*TSR1*), translation elongation factor 1-alpha gene region (*EF1A*), calmodulin gene (*CaM*), partial γ-actin (*ACT*), DNA replication licensing factor (*MCM7*), translation elongation factor 3 (*TEF3*) and 60S ribosomal protein L10 (*RP 60S L1*) etc. (Table 1). The amplification reactions were performed in 25 μl final volumes consisted of 2 µl DNA template (10 ng/μl), 1 µl forward primer (10 µM), 1 µl reverse primer (10 µM), 12.5 µl 2× SanTaq PCR Master Mix (containing Taq polymerase, dNTP and Mg^2+^; Sangon Biotech Co., Ltd, Shanghai, China) and 8.5 µl sterile water. The PCR was run using a T100 (Bio-Rad, California, USA) Thermal Cycler and the resulting amplified PCR products were sequenced in both directions using PCR primers. After amplification, the PCR products were visualised on a 1% agarose gel stained with ethidium bromide and the positive PCR products were then sent for sequencing to Sangon Biotech (Shanghai, China). The primer pairs and amplification conditions for each of the above-mentioned gene regions are provided in Table 1. All of the new sequences generated were deposited to GenBank (Suppl. material 1).

**Table 1.** Sequences of primers were used for the amplification of molecular markers in this study.

Molecular marker	Primer name	Primer sequence (5´-3´)	Optimised PCR protocols	Reference
*ACT*	ACT-512F	ATGTGCAAGGCCGGTTTCGC	94 °C: 5 min, (94 °C: 30 s, 55 °C: 50 s, 72 °C: 1 min) × 35 cycles 72 °C: 10 min	Carbone and Kohn (1999)
	ACT-783R	TACGAGTCCTTCTGGCCCAT		
*CaM*	CF1	GCCGACTCTTTGACYGARGAR	94 °C: 5 min, (94 °C: 30 s, 55 °C: 30 s, 72 °C: 1 min) × 35 cycles, 72 °C: 10 min	Peterson et al. (2005)
	CF4	TTTYTGCATCATRAGYTGGAC		
	CAL-CL1	GARTWCAAGGAGGCCTTCTC	94 °C: 5 min, (94 °C: 45 s, 55 °C: 45 s, 72 °C: 1 min) × 35 cycles, 72 °C: 10 min	O’Donnell et al. (2000)
	CAL-CL2A	TTTTTGCATCATGAGTTGGAC		
*EF1A*	983F	GCYCCYGGHCAYCGTGAYTTYAT	94 °C: 5 min, (94 °C: 30 s, 58 °C: 1 min 20 s, 72 °C: 1 min) × 35 cycles, 72 °C: 10 min	Rehner and Buckley (2005)
	2218R	ATGACACCRACRGCRACRGTYTG		
	EF-1	ATGGGTAAGGARGACAAGAC	94 °C: 5 min, (94 °C: 45 s, 55 °C: 45 s, 72 °C: 1 min) × 35 cycles, 72 °C: 10 min	O’Donnell et al. (1998)
	EF-2	GGARGTACCAGTSATCATG		
ITS	ITS1	TCCGTAGGTGAACCTGCG	94 °C: 5 min, (94 °C: 30 s, 51 °C: 50 s, 72 °C: 45 s) × 35 cycles, 72 °C: 10 min	White et al. (1990)
	ITS4	TCCTCCGCTTATTGATATGC		
LSU	LR0R	ACCCGCTGAACTTAAGC	94 °C: 5 min, (94 °C: 30 s, 51 °C: 1 min, 72 °C: 2 min) × 35 cycles, 72 °C: 10 min	Vilgalys and Hester (1990)
	LR7	TACTACCACCAAGATCT		
*MCM7*	Mcm7-709f	ACNMGNGTNTCVGAYGTHAARCC	94 °C: 5 min, (94 °C: 1 min, 55 °C: 1 min, 72 °C: 1 min 40 s) × 35 cycles 72 °C: 10 min	Schmitt et al. (2009)
	Mcm7-1348r	GAYTTDGCNACNCCNGGRTCWCCCAT		
*RP 60S L1*	60S-506F	GHGACAAGCGTTTCTCNGG	94 °C: 5 min, (94 °C: 45 s, 55 °C: 50 s, 72 °C: 1 min) × 35 cycles 72 °C: 10 min	Stielow et al. (2015)
	60S-908R	CTTVAVYTGGAACTTGATGGT		
*RPB2*	fRPB2-5F	GAYGAYMGWGATCAYTTYGG	94 °C: 5 min, (94 °C: 30 s, 54 °C: 40 s, 72 °C: 1 min 20 s) × 35 cycles, 72 °C: 10 min	Liu et al. (1999)
	RPB2-7cR	CCCATRGCTTGYTTRCCCAT		
	fRPB2-7cF	ATGGGYAARCAAGCYATGGG	94 °C: 5 min, (94 °C: 1 min, 50 °C: 2 min, 72 °C: 2 min 10 s) × 35 cycles 72 °C: 10 min	Liu et al. (1999)
	RPB2-3053bR	TGRATYTTRTCRTCSACCAT		Reeb et al. (2004)
*TEF3*	EF3-3185F	TCYGGWGGHTGGAAGATGAAG	94 °C: 5 min, (94 °C: 45 s, 55 °C: 50 s, 72 °C: 1 min) × 35 cycles 72 °C: 10 min	Stielow et al. (2015)
	EF3-3188F	GGHGGHTGGAAGATGAAG		
	EF3-3538R	YTTGGTCTTGACACCNTC		
	EF3-3984R	TCRTAVSWGTTCTTGAACTT		
*TSR1*	F1526Pc	GARTAYCCBCARTCNGAGATGT	94 °C: 5 min, (94 °C: 30 s, 50 °C: 30 s, 72 °C: 1 min) × 35 cycles, 72 °C: 10 min	Houbraken et al. (2011b)
	R2434	ASAGYTGVARDGCCTTRAACCA		
*TUB*	BT-2a	GGTAACCAAATCGGTGCTGCTTTC	94 °C: 5 min, (94 °C: 30 s, 58 °C: 50 s, 72 °C: 1 min) × 35 cycles 72 °C: 10 min	Glass and Donaldson (1995)
	Bt-2b	ACCCTCAGTGTAGTGACCCTTGGC		
	TUB2Fd	GTBCACCTYCARACCGGYCARTG	94 °C: 5 min, (94 °C: 30 s, 52 °C: 30 s, 72 °C: 30 s) × 35 cycles 72 °C: 10 min	Woudenberg et al. (2009)
	TUB4Fd	CCRGAYTGRCCRAARACRAAGTTGTC		
	Btub526_F	CGAGCGYATGAGYGTYTACTT	94 °C: 5 min, (94 °C: 30 s, 53 °C: 45 s, 72 °C: 1 min 30 s) × 35 cycles 72 °C: 10 min	Jewell and Hsiang (2013)
	Btub1332_R	TCATGTTCTTGGGGTCGAA		

### ﻿Phylogenetic analysis

The collation of sequences (including name simplification and renaming) was performed using TBtools software (Chen et al. 2020). The sequence set was aligned by MAFFT v.7.037v (Katoh and Standley 2013), with sequence editing and trimming implemented in MEGA v.6.06 (Tamura et al. 2013). The “Concatenate Sequence” function in PhyloSuite v.1.16 (Zhang et al. 2020a) was used for the concatenation of loci. All concatenate data are provided in Suppl. material 3. For the phylogenetic construction of each loci dataset, both the Maximum Likelihood (ML) and the Bayesian Inference (BI) methods were used. The best-fit substitution model was selected using the Akaike Information Criterion correction (AICc), in ModelFinder (Kalyaanamoorthy et al. 2017). The ML analysis was implemented in IQ-TREE v.1.6.11 (Nguyen et al. 2015) with 10^4^ bootstrap tests using the ultrafast algorithm (Minh et al. 2013). For Bayesian Inference, MrBayes v.3.2 (Ronquist et al. 2012) was used and Markov Chain Monte Carlo (MCMC) simulations were run for 5 × 10^7^ generations with a sampling frequency of every 10^3^ generations and a burn-in of 25%. The above analyses were carried out in PhyloSuite v.1.16 (Zhang et al. 2020a). Tracer v.1.7 (Rambaut et al. 2018) was used to assess the convergence and effective sampling between all runs; the obtained effective sample size (ESS) values were all greater than 200, indicating that effective sampling occurred. The final trees were visualised in FigTree 1.4.23 and edited in Microsoft Paint.

**Figure 1.**
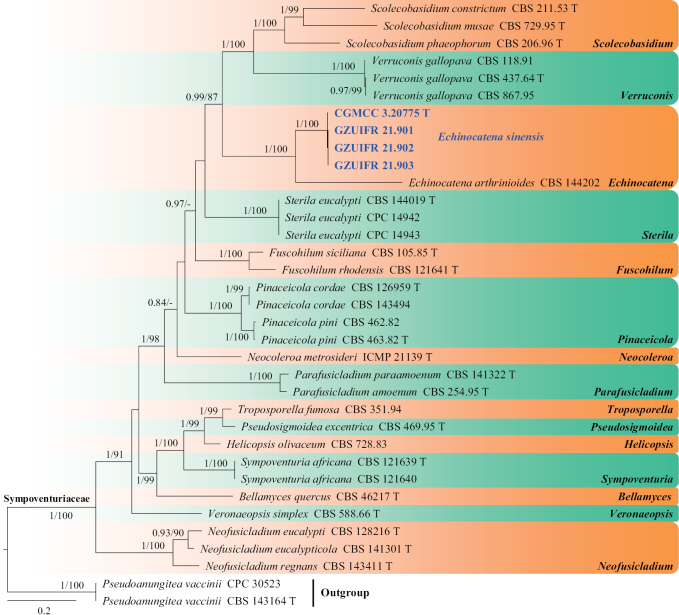
Concatenated phylogeny of the ITS, LSU, *EF1A*, *TUB* and *RPB2* gene regions of species in Sympoventuriaceae. Thirty-five strains are used. The tree is rooted with *Pseudoanungiteavaccinii* (CPC 30523) and *P.vaccinii* (CBS 143164). The tree topology of the BI was similar to the ML analysis. Bayesian posterior probability (≥ 0.8) and ML bootstrap values (≥ 80%) are indicated along branches (PP/ML). Novel species are in blue and bold font and “T” indicates type derived sequences.

## ﻿Taxonomy

### ﻿Dothideomycetes O.E. Erikss. & Winka.

**Venturiales Yin. Zhang, C.L. Schoch & K.D. Hyde.**

**Sympoventuriaceae Yin. Zhang, C.L. Schoch & K.D. Hyde.**

***Echinocatena* R. Campb. & B. Sutton.**

The establishment of the genus *Echinocatena* dates back to 1977, with *E.arthrinioides* being the only valid species to date. This species was isolated from the apoplastic leaves of an unknown plant (Campbell and Sutton 1977).

#### ﻿ Echinocatena sinensis

Taxon classificationFungiVenturialesSympoventuriaceae

Zhi.Y. Zhang, Y.F. Han & Z.Q. Liang sp. nov.

F3E24229-547E-5B16-A9FA-FFEDF9FD7401

: 844151

Fig. 2

##### Etymology.

The epithet refers to the locality where the type specimen was found, China.

**Figure 2.**
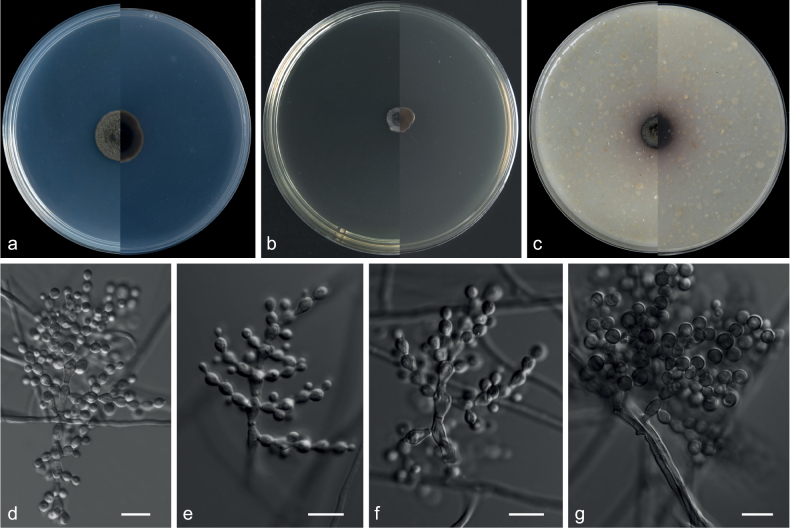
*Echinocatenasinensis* (from ex-holotype CGMCC 3.20775) **a–c** upper and reverse views of cultures on PDA, MEA and OA 14 d after inoculation **d–g** phialides and conidia. Scale bars: 10 µm (**d–g**).

##### Type.

China: Guangxi Zhuang Autonomous Region, Baise City, Peninsula Park 23°89'96"N, 106°63'29"E, from soil, 30 Aug 2019, Z.Y. Zhang (HMAS 351873 holotype designated here, ex-type living culture CGMCC 3.20775 = GZUIFR 21.900); ibid., GZUIFR 21.901.

##### Description.

***Culture characteristics*** (14 days at 25 °C): ***Colony on PDA*** 16–18 mm diam. after 14 d at 25 °C, dark olive green (2F2), flat, texture velvety, nearly round, margin entire; reverse dark slate grey (3F2). ***Colony on MEA*** 8–9 mm diam., dark slate grey (3F1), convex, texture velvety, irregularity, margin entire; reverse dark slate grey (3F2). ***Colony on OA*** 10–12 mm diam., dark slate grey, flat (3F2), texture velvety, nearly round, margin entire, soluble pigments brown to pale red exudates absent; reverse dark pink (12F1).

***Hyphae*** branched, septate, hyaline, smooth, 1.0–3.5 μm diam. ***Conidiophores*** erect or with an acute angle to the axis near the apex, solitary, unbranched, 9.5–49.0 × 1.0–5.0 µm, hyaline, smooth, 1–8-septate, straight to flexuous. ***Conidiogenous cells*** in simple or branched acropetal chains, 4.0–8.5 × 3.0–5.0 µm, separated by thick, dark brown, refractive septa, appearing like a separating cell, pale brown, doliiform to cylindrical, constricted at the septa, polyblastic, integrated with 3–5 conidiogenous loci. ***Conidia*** solitary, pyriform, sometimes spherical, aseptate, smooth, brown, 3.5–7.0 × 3.5–7.0 µm (av. 4.9 × 5.3 μm, n = 50). ***Sexual morph*** not observed.

##### Additional specimens examined.

China: Guangxi Zhuang Autonomous Region, Baise City, Youjiang Campus of Baise University 23°89'10"N, 106°60'86"E, from soil, 30 Aug 2019, Z.Y. Zhang, GZUIFR 21.902, ibid., GZUIFR 21.903.

##### Notes.

Currently, one species is accepted in *Echinocatena* (Campbell and Sutton 1977; Shen et al. 2020). The phylogenetic analyses, based on the combined ITS, LSU, *EF1A*, *TUB* and *RPB2* dataset, indicate *Echinocatenasinensis* and *E.arthrinioides* group in a distinct clade (Fig. 1). The morphology of *E.sinensis* is very similar to *Echinocatena* in having straight to ﬂexuous conidiophores, polyblastic conidiogenous cells, spherical, aseptate conidia (Campbell and Sutton 1977). *Echinocatenasinensis* can be distinguished from *E.arthrinioides* by its conidia that are smooth and pyriform in shape (Campbell and Sutton 1977), as well as by the low sequence similarity between the two species (ITS: 312/403, 77.4% similarity, 25 gaps; LSU: 787/827, 95.2% similarity, 4 gaps).

### ﻿Eurotiomycetes O.E. Erikss. & Winka

**Eurotiales G.W. Martin ex Benny & Kimbr.**

**Aspergillaceae Link**

***Aspergillus* P. Micheli ex Haller**

Micheli (1729) erected the genus *Aspergillus*, whose members can be identified by their aspergillum-like spore-bearing structure (Samson et al. 2014). Currently, the genus *Aspergillus* comprises six subgenera and 27 sections (Houbraken et al. 2020). In this study, two new species are described: *A.cylindricus* and *A.doliiformis*.

#### ﻿ Aspergillus cylindricus

Taxon classificationFungiEurotialesAspergillaceae

Zhi.Y. Zhang, Y.F. Han & Z.Q. Liang sp. nov.

0766958C-A2AD-5B6F-8182-65A35DD508CB

: 844152

Fig. 4

##### Etymology.

Referring to the cylindrical phialides.

##### Type.

China: Shanghai Municipality, Ruijin Hospital, Shanghai Jiao Tong University School of Medicine 31°21'29"N, 121°46'75"E, from soil, 15 Aug 2019, Z.Y. Zhang (HMAS 351868 holotype designated here, ex-type living culture CGMCC 3.20771 = GZUIFR 21.887); ibid., GZUIFR 21.888.

##### Description.

***Culture characteristics*** (14 d at 25 °C): ***Colony on PDA*** 12–13 mm diam., pinkish-white (10A1), velvety to ﬂoccose, margin slightly undulate; reverse chrome orange (6A6) to yellowish-white (2A2) from centre to margin. ***Colony on MEA*** 15–17 mm diam., bluish-white (1A2), felty, margin dentate; reverse mandarin orange (6B8). ***Colony on OA*** 26–27 mm diam., white (4A1), velvety to ﬂoccose, margin slightly undulate; reverse brownish-yellow (5C8) to grey (5C1) from centre to margin.

***Conidiophores*** solitary phialides borne laterally or terminally on vegetative hyphae, sometimes occurring in branched hyphal resembling branched conidiophores. ***Phialides*** mono- to polyphialidic, hyaline, cylindrical to lageniform, sometimes curved irregularly, swollen towards the base or above the mid-section, neck cylindrical or broadly tapering, sometimes extending sympodially from the neck, 2.0–21.0 × 1.0–5.0 µm. ***Conidia*** borne solitary or in chains with pronounced connectors, hyaline, smooth or roughened, ellipsoid to subglobose, pyriform, 3.0–7.5 × 2.5–5.5 µm (av. 5.3 × 4.5 μm, n = 50). ***Sexual morph*** not observed.

##### Additional specimens examined.

China: Shanghai Municipality, South Campus of Fudan University 31°29'30"N, 121°50'03"E, soil, 16 Aug 2019, Z.Y. Zhang, GZUIFR 21.889.

##### Notes.

**Figure 3.**
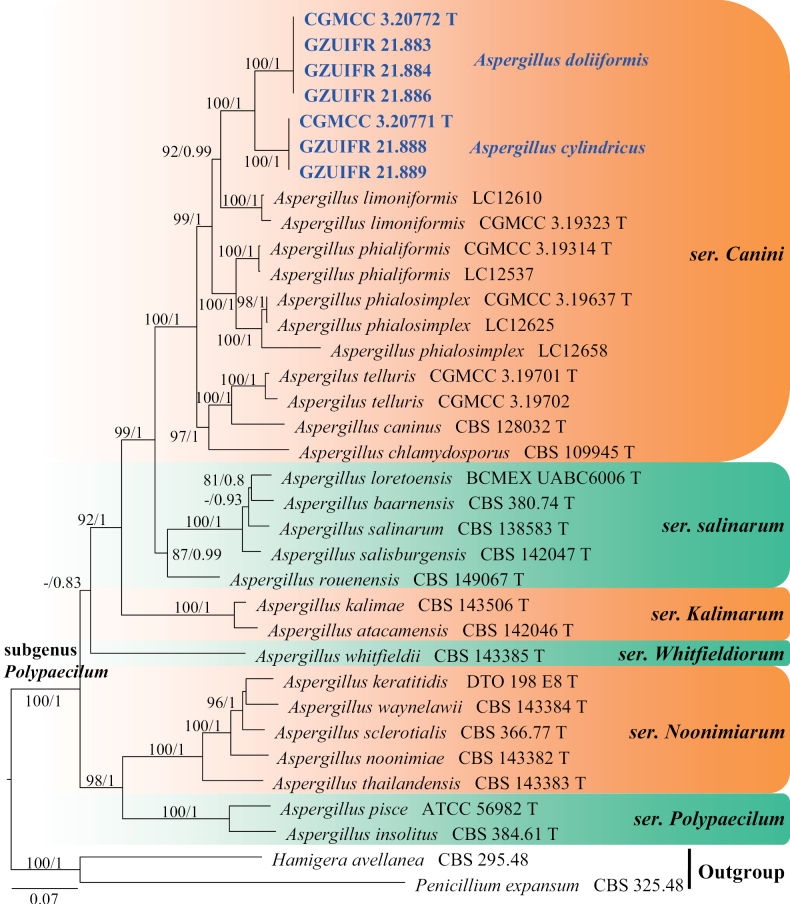
Concatenated phylogeny of the ITS, *TUB*, *CaM*, *RPB2* and *TSR1* gene regions of species in *Aspergillus* from subgenus Polypaecilum. Thirty-five strains are used. The tree is rooted in *Hamigeraavellanea* (CBS 295.48) and *Penicilliumexpansum* (CBS 325.48). The tree topology of the BI was similar to the ML analysis. Bayesian posterior probability (≥ 0.8) and ML bootstrap values (≥ 80%) are indicated along branches (PP/ML). Novel species are in blue and bold font and “T” indicates type derived sequences.

**Figure 4.**
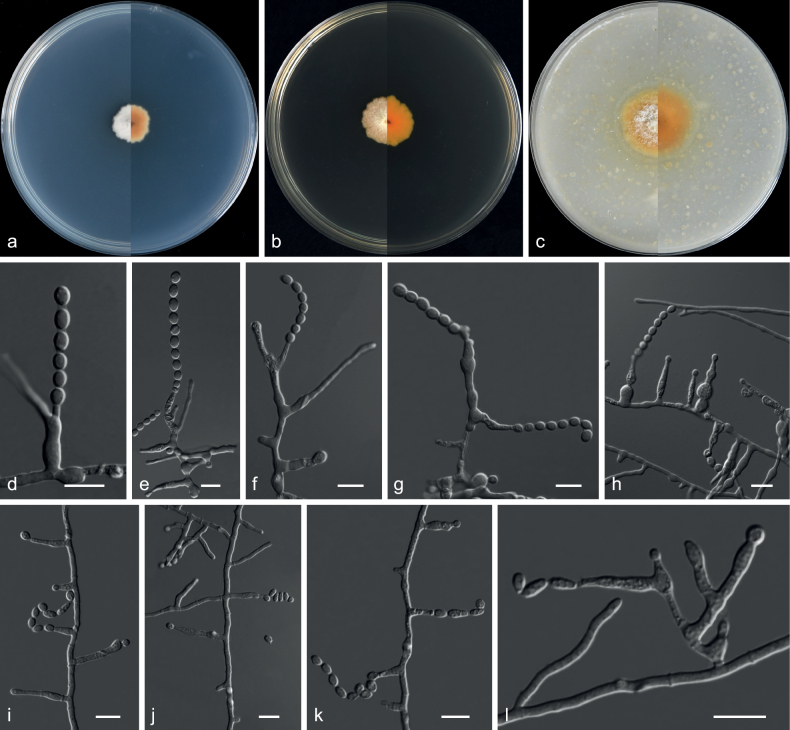
*Aspergilluscylindricus* (from ex-holotype CGMCC 3.20771) **a–c** upper and reverse views of cultures on PDA, MEA and OA 14 d after inoculation **d–l** phialides and conidia. Scale bars: 10 µm (**d–l**).

Phylogenetic and morphological data (Figs 3, 4) support our isolates CGMCC 3.20771, GZUIFR 21.888 and GZUIFR 21.889 as new species of subgenus Polypaecilum, series *Canini*. *Aspergilluscylindricus* is phylogenetically closely related to *A.doliiformis* and *A.limoniformis*. However, they can be distinguished by their sequence similarity (94% 498/531; 99% 846/853; 88% 394/448; 92% 767/834; 92% 723/788 similarity of ITS, LSU, *TUB*, *RPB2* and *TSR1* in *A.limoniformis*CGMCC 3.19323; 94% 524/554, 99% 1337/1341, 91% 419/458, 94% 1007/1069, 92% 772/843, 91% 629/692 similarity of ITS, LSU, *TUB*, *RPB2*, *TSR1* and *CaM* in *A.doliiformis*CGMCC 3.20772). Morphologically, the conidia of *A.limoniformis* are limoniform or subglobose, rather than ellipsoid to subglobose, pyriform in *A.cylindricus* (Zhang et al. 2021a). On the other hand, *A.cylindricus* has longer phialides than *A.limoniformis* (2.0–21.0 µm vs. 4.0–10.0 µm) (Zhang et al. 2021a). Furthermore, conidia of *A.doliiformis* are lantern, subglobose to globose, obpyriform rather than ellipsoid to subglobose, pyriform in *A.cylindricus*. The conidiophores of *A.cylindricus* are sometimes occurring in branched hyphae resembling branched conidiophores, while the conidiophores of *A.doliiformis* are borne laterally or terminally on vegetative hyphae (see description of *A.doliiformis*).

#### ﻿ Aspergillus doliiformis

Taxon classificationFungiEurotialesAspergillaceae

Zhi.Y. Zhang, Y.F. Han & Z.Q. Liang sp. nov.

7933C06B-5492-51A3-9828-A0DCDF4858A4

: 844153

Fig. 5

##### Etymology.

Referring to the lantern shape of conidia.

##### Type.

Hainan Province, Haikou City, Haidian Campus of Hainan University 20°05'76"N, 110°32'91"E, from soil, 28 Aug 2019, Z.Y. Zhang (HMAS 351867 holotype designated here, ex-type living culture CGMCC 3.20772 = GZUIFR 21.885); ibid., GZUIFR 21.883.

**Figure 5.**
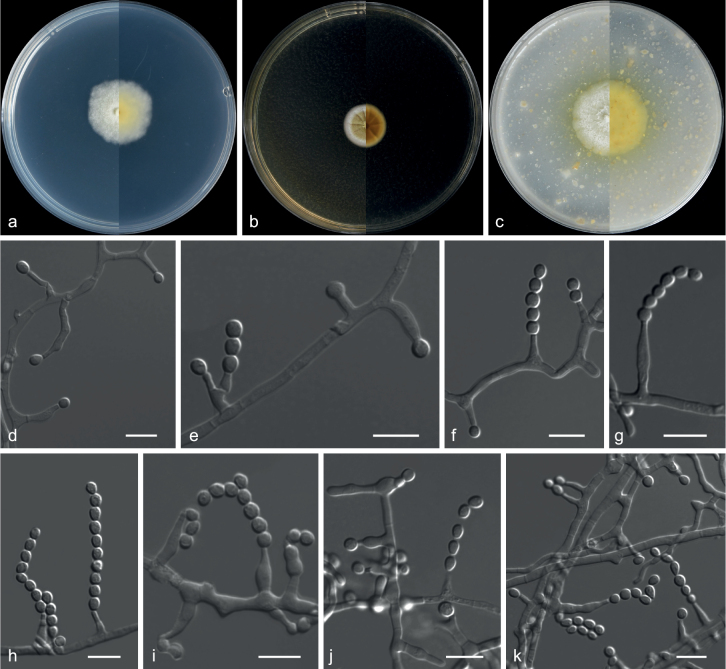
*Aspergillusdoliiformis* (from ex-holotype CGMCC 3.20772) **a–c** upper and reverse views of cultures on PDA, MEA and OA 14 d after inoculation **d–k** phialides and conidia. Scale bars: 10 µm (**d–k**).

##### Description.

***Culture characteristics*** (14 d at 25 °C): ***Colony on PDA*** 23–26 mm diam., white (1A1), felty, fluffy, margin slightly undulate; reverse pale yellow (1A3) to white (1A1) from centre to margin. ***Colony on MEA*** 16 mm diam., white (1A1) at margin, olive yellow (2D8) at centre, felty, margin entire; reverse oak brown (5D6) to brownish-grey (5D2) from centre to margin. ***Colony on OA*** 26–27 mm diam., white (1A1), flocculent, margin entire, producing a diffusible faint yellow pigment; reverse greyish-yellow (2B4).

***Hyphae*** hyaline, septate, smooth, branched, 1.0–3.5 μm wide. ***Conidiophores*** solitary phialides borne laterally or terminally on vegetative hyphae. ***Phialides*** mono- to polyphialidic, hyaline, ampulliform, campaniform, cylindrical or tapering with an enlarged base, sometimes curved irregularly, occasionally branched, neck cylindrical or broadly tapering, sometimes extending sympodially from the necks, 2.5–18.0 × 1.0–5.0 µm. ***Conidia*** borne solitary or formed in long chains, hyaline, smooth or roughened, lantern, subglobose to globose, obpyriform, 3.0–5.0 × 2.5–4.5 µm (av. 4.2 × 3.4 μm, n = 50). ***Sexual morph*** not observed.

##### Additional specimens examined.

China: Hainan Province, Haikou City, Hainan General Hospital 20°00'57"N, 110°28'78"E, from soil, 28 Aug 2019, Z.Y. Zhang, GZUIFR 21.884; Haikou People’s Park, soil, 28 Aug 2019, Z.Y. Zhang, GZUIFR 21.886.

##### Notes.

*Aspergillusdoliiformis* represents a new lineage within the subgenus Polypaecilum, series *Canini*, forming a fully supported clade (ML = 100%; PP = 1.0). *A.doliiformis* is phylogenetically closely related to *A.limoniformis* and *A.cylindricus*. However, *A.doliiformis* can be distinguished from *A.limoniformis* by their sequence similarity (93% 502/539; 99% 847/853; 87% 370/423; 91% 762/834; 92% 724/791 similarity of ITS, LSU, *TUB*, *RPB2* and *TSR1* in *A.limoniformis*CGMCC 3.19323). Morphologically, the conidia of *A.limoniformis* are limoniform or subglobose, rather than lantern, subglobose to globose, obpyriform in *A.doliiformis* (Zhang et al. 2021a). In addition, the distinction between *A.doliiformis* and *A.cylindricus* is shown in the notes of *A.cylindricus*.

### ﻿*Penicillium* Link

The genus *Penicillium* was established in 1809 (Link 1809) and its members are widespread, occurring in abundance in soil, air, indoor environments and contaminated foods (Visagie et al. 2014; Barbosa et al. 2018). Currently, this genus comprises two subgenera and 32 sections (Houbraken et al. 2020). In the present study, one new species is described: *P.fujianense*.

#### ﻿ Penicillium fujianense

Taxon classificationFungiEurotialesAspergillaceae

Zhi.Y. Zhang, Y.F. Han & Z.Q. Liang sp. nov.

A4B65618-23AE-5577-850C-DA1C842559BA

: 844154

Fig. 7

##### Etymology.

Referring to its origin, isolated from Fujian Province, China.

##### Type.

China: Fujian Province, Xiamen City, Jimei University 24°58'25"N, 118°09'46"E, soil, 18 Aug 2019, Z.Y. Zhang (HMAS 351866 holotype designated here, ex-type living culture CGMCC 3.20781 = GZUIFR 21.880).

##### Description.

***Culture characteristics*** (14 d at 25 °C): ***Colony on PDA*** 18–21 mm diam., white, mycelium inconspicuous, texture velvety, sporulation dense, conidial area dark green (28F8), margin irregular, soluble pigments and exudates absent; reverse milk white (1A2). ***Colony on MEA*** 18–25 mm diam., white, texture velvety, sporulation dense, conidial area greenish-white (28A2), margin irregular, soluble pigments and exudates absent; reverse orange (5A7). ***Colony on OA*** 72 mm diam., surrounded by an orange halo, mycelium white, texture velvety, sporulation dense, conidia area masse dark green (26F4), margins entire, soluble pigments light brown, exudates absent; reverse butter yellow (4A5).

***Hyphae*** hyaline, septate, smooth, branched, 1.0–4.0 μm wide. ***Conidiophores*** biverticillate and occasionally with an additional divergent branch, stipes variable in length, 10–74 µm long, smooth, 2.0–3.5 µm wide. ***Metulae*** divergent, 2–6 per stipe, slightly inflated at the apex, 10.5–20 × 2.0–4.0 µm. ***Phialides*** ampulliform to cylindrical with a short neck, stout, 4.0–11.0 × 2.0–3.5 µm. ***Conidia*** subglobose to globose, ellipsoidal, finely roughened, 2.0–4.5 × 2.0–3.5 µm (av. 3.8 × 3.2 μm, n = 50). ***Sexual morph*** not observed.

##### Additional specimens examined.

China: Fujian Province, Xiamen City, Wuyuanbay Wetland Park 24°51'52"N, 118°17'48"E, soil, 19 Aug 2019, Z.Y. Zhang, GZUIFR 21.881, ibid., GZUIFR 21.882.

##### Notes.

**Figure 6.**
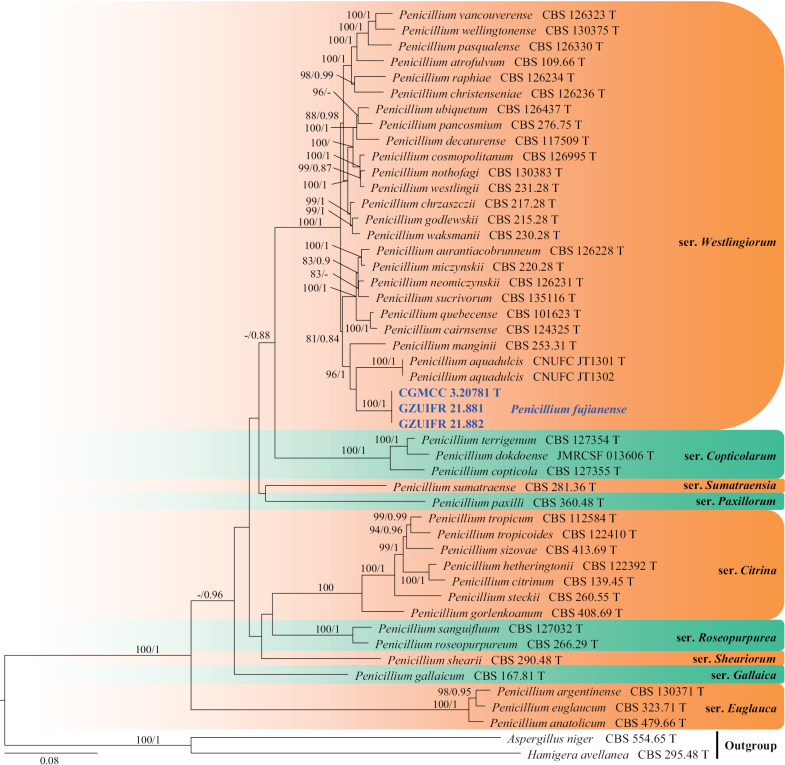
Concatenated phylogeny of the ITS, *TUB*, *CaM*, *RPB2* and *TSR1* gene regions of species in *Penicillium* from section Citrina. Forty-eight strains are used. The tree is rooted in *Aspergillusniger* (CBS 554.65) and *Hamigeraavellanea* (CBS 295.48). The tree topology of the BI was similar to the ML analysis. Bayesian posterior probability (≥ 0.8) and ML bootstrap values (≥ 80%) are indicated along branches (PP/ML). Novel species are in blue and bold font and “T” indicates type derived sequences.

*Penicilliumfujianense* represents a new lineage in the subgenus Aspergilloides, section Citrina and *Westlingiorum* series, forming a strongly supported clade (ML = 100%; PP = 1.0), closely related to *P.manginii* and *P.aquadulcis* (Fig. 6). However, *P.fujianense* distinguished from *P.manginii* by its absent sclerotia (Houbraken et al. 2011a). *P.fujianense* can be distinguished from *P.aquadulcis* by their conidial shape and size (subglobose to globose, ellipsoidal, 3.8 × 3.2 µm in *P.fujianense*; globose to subglobose, 2–2.5 µm in *P.aquadulcis*) (Thuong et al. 2021). Furthermore, in a comparison of ITS, *BenA* and *CaM* nucleotides, *P.fujianense* (Type strain CGMCC 3.20781) has 98%, 94% and 92% similarity, in ITS (504/512 bp, one gap), *BenA* (401/427, no gap) and *CaM* (516/559, three gaps), which is different from *P.manginii* (Type strain CBS 253.31). Additionally, *P.fujianense* (Type strain CGMCC 3.20781) has 97%, 93% and 91% similarity, in ITS (527/543 bp, four gaps), BenA (410/441, no gap) and CaM (545/598, 15 gaps), which is different from *P.aquadulcis* (Type strain CNUFC JT1301). DNA sequencing and multigene phylogeny provide the most reliable identification of this species.

**Figure 7.**
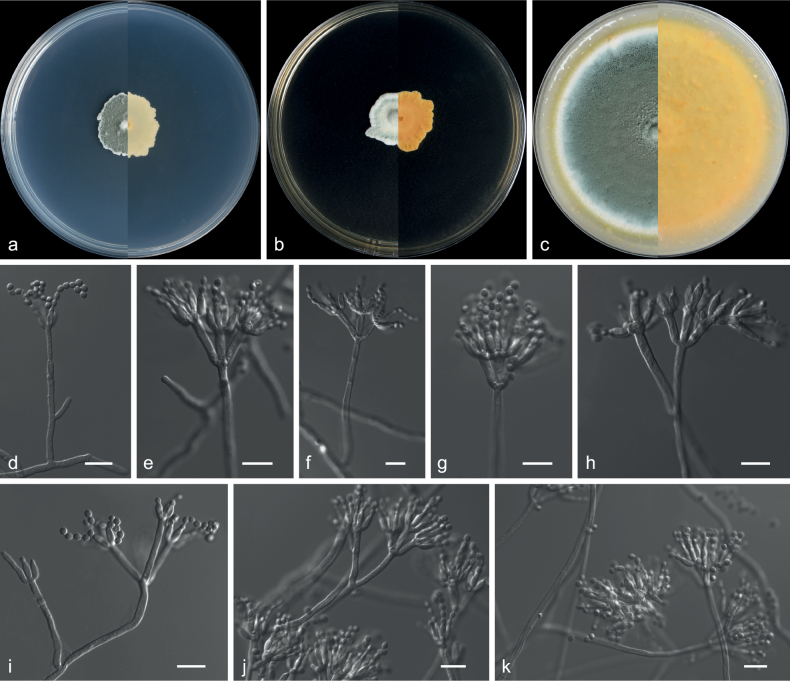
*Penicilliumfujianense* (from ex-holotype CGMCC 3.20781) **a–c** upper and reverse views of cultures on PDA, MEA and OA 14 d after inoculation **d–k** phialides and conidia. Scale bars: 10 µm (**d–k**).

### ﻿*Talaromyces* C.R. Benj.

The genus *Talaromyces* was erected in 1955 to accommodate sexual species in the genus *Penicillium* (Benjamin 1955). Members of the genus *Talaromyces* have a worldwide distribution and colonises many substrates, predominantly soil (Sun et al. 2020). Currently, the genus *Talaromyces* includes eight sections, *Bacillispori*, *Helici*, *Islandici*, *Purpurei*, *Tenues*, *Subinflati*, *Talaromyces* and *Trachyspermi* (Yilmaz et al. 2014; Sun et al. 2020). In this study, three new species are described *T.guiayngensis*, *T.jiangxiensis* and *T.paecilomycetoides*.

#### ﻿ Talaromyces guiyangensis

Taxon classificationFungiEurotialesAspergillaceae

Zhi.Y. Zhang, Y.F. Han & Z.Q. Liang sp. nov.

B84945CD-5AF3-51FE-9F81-D038A5769032

: 844155

Fig. 9

##### Etymology.

Referring to its origin, isolated from Guiyang City, China.

##### Type.

China: Guizhou Province, Guiyang City, Qianlingshan Park 26°59'03"N, 106°69'57"E, soil, 13 Sept 2019, Z.Y. Zhang (HMAS 351869 holotype designated here, ex-type living culture CGMCC 3.20782 = GZUIFR 21.890).

##### Description.

***Culture characteristics*** (14 d at 25 °C): ***Colony on PDA*** 32–34 mm diam., moderately deep, mycelium white, primrose, velutinous, planar, margins entire and slightly undulate, sporulation dense, conidial area dark green (29F8), soluble pigments and exudates absent; reverse light yellow (4A5) to rust brown (6E8) from margin to the centre. ***Colony on MEA*** 34–38 mm diam., moderately deep, sunken at the centre, mycelium white, texture velutinous, sporulation dense, conidial area dark green (29F8), soluble pigments and exudates absent; reverse pale green (30A3). ***Colony on OA*** 32–33 mm diam., moderately deep, mycelium white, margins high, narrow, entire, white (1A1), conidial area grey (29F1), soluble pigments and exudates absent; reverse pastel yellow (1A4).

***Hyphae*** hyaline, septate, smooth, branched, 1.0–3.0 μm wide. ***Conidiophores*** smooth, biverticillate, stipes smooth, bearing terminal biverticillate penicillin. ***Metulae*** 3–5, divergent, 8.5–13.5 × 2.0–3.0 μm. ***Phialides*** 2–5, acerose, 9.0–13.0 × 1.5–2.5 μm, with long gradually tapering collula. ***Conidia*** spiny, fusiform, pyriform, 3.0–6.0 × 2.5–3.0 μm (av. 4.5 × 2.8 μm, n = 50). ***Sexual morph*** not observed.

##### Additional specimens examined.

China: Guizhou Province, Guiyang City, North Campus of Guizhou University 26°44'37"N, 106°67'46"E, soil, 13 Sept 2019, Z.Y. Zhang, GZUIFR 21.891.

##### Notes.

**Figure 8.**
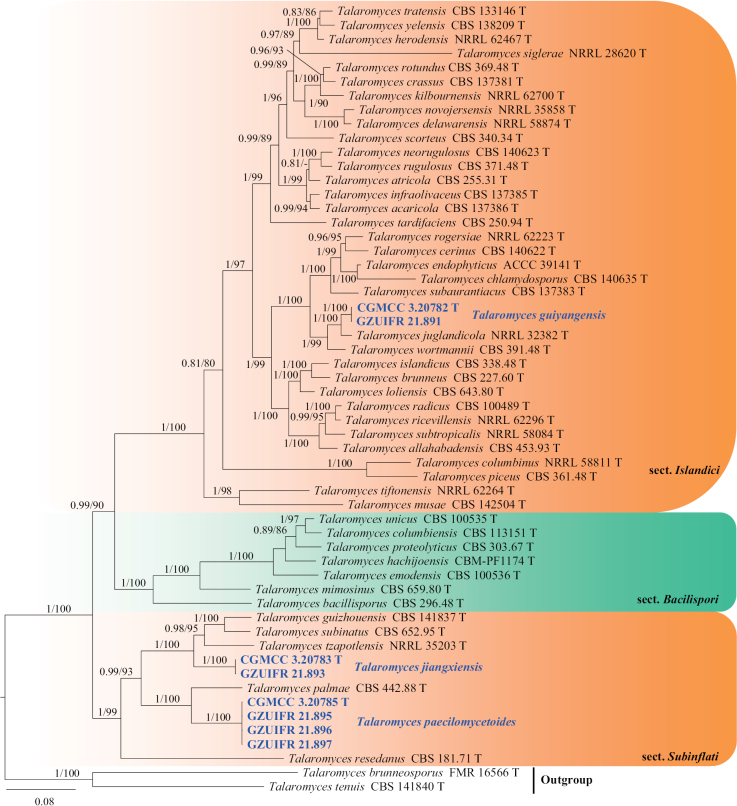
Concatenated phylogeny of the ITS, *TUB*, *CaM* and *RPB2* gene regions of species in *Talaromyces* from sections *Islandici*, *Bacillispori* and *Subinflati*. Fifty-six strains are used. The tree is rooted with *Talaromycesbrunneosporus* (FMR 16566) and *T.tenuis* (CBS 141840). The tree topology of the BI was similar to the ML analysis. Bayesian posterior probability (≥ 0.8) and ML bootstrap values (≥ 80%) are indicated along branches (PP/ML). Novel species are in blue and bold font and “T” indicates type derived sequences.

*Talaromycesguiyangensis* represents a new lineage in the section Islandici, forming a strongly-supported clade (ML = 100%; PP = 1.0), closely related to *T.juglandicola* and *T.wortmannii* (Fig. 8). However, *T.guiyangensis* differs from *T.juglandicola* by its fusiform, pyriform conidia and does not produce exudate or droplets on PDA or MEA (Peterson and Jurjević 2017). In addition, *Talaromyceswortmannii* differs from *T.guiyangensis* in the presence of ascomata and ascospores, which are not observed in *T.guiyangensis*. Furthermore, the conidia of *T.wortmannii* are ellipsoidal, whereas those of *T.guiyangensis* are fusiform or pyriform (Yilmaz et al. 2014).

**Figure 9.**
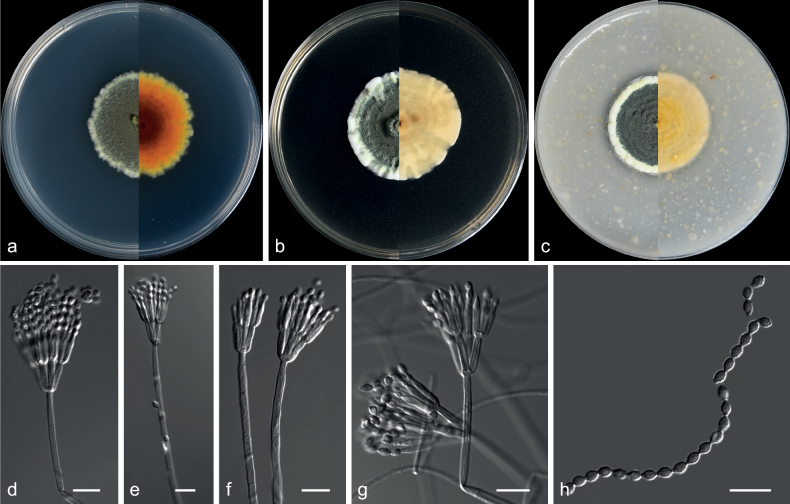
*Talaromycesguiyangensis* (from ex-holotype CGMCC 3.20782) **a–c** upper and reverse views of cultures on PDA, MEA and OA 14 d after inoculation **d–g** phialides and conidia **h** conidia chain. Scale bars: 10 µm (**d–h**).

#### ﻿ Talaromyces jiangxiensis

Taxon classificationFungiEurotialesAspergillaceae

Zhi.Y. Zhang, Y.F. Han & Z.Q. Liang sp. nov.

D74D8997-6B03-582E-B92E-229823DF3203

: 844156

Fig. 10

##### Etymology.

Referring to its origin, isolated from Nanchang City, Jiangxi Province, China.

##### Type.

China: Jiangxi Province, Nanchang City, Nanchang People’s Park 28°68'12"N, 115°91'35"E, soil, 13 Aug 2019, Z.Y. Zhang (HMAS 351870 holotype designated here, ex-type living culture CGMCC 3.20783 = GZUIFR 21.892); ibid., GZUIFR 21.893.

##### Description.

***Culture characteristics*** (14 d at 25 °C): ***Colony on PDA*** 50–51 mm diam., moderately deep, plane, mycelium white, planar, sporulation dense, margins entire, slightly undulate, conidial area dark green (30F3), soluble pigments and exudates absent; reverse white (30A3). ***Colony on MEA*** 26–33 mm diam., moderately deep, mycelium pale golden rod at the centre, white at the edge, texture velvety, margins irregular, sporulation dense, conidia area yellowish-white (1A2), soluble pigments and exudates absent; reverse greyish-yellow (2C3). ***Colony on OA*** 32–33 mm diam., moderately deep, mycelium white, texture velutinous, margins low, narrow, irregular, sporulation moderately dense, conidia masse greenish-grey (30F2), soluble pigments and exudates absent; reverse pastel green (30A4).

**Figure 10.**
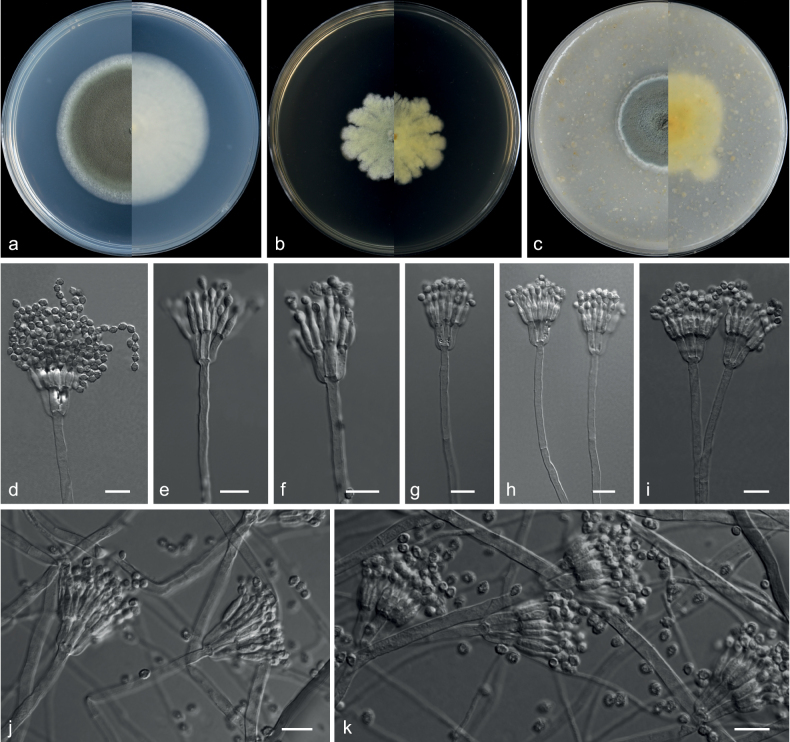
*Talaromycesjiangxiensis* (from ex-holotype CGMCC 3.20783) **a–c** upper and reverse views of cultures on PDA, MEA and OA 14 d after inoculation **d–k** phialides and conidia. Scale bars: 10 µm (**d–k**).

***Hyphae*** hyaline, septate, smooth, branched, 1.0–4.5 μm wide. ***Conidiophores*** smooth, biverticillate, stipes smooth, bearing terminal biverticillate penicillin. ***Metulae*** 3–6, divergent, 8.0–13.5 × 2.0–4.0 μm. ***Phialides*** 3–6, acerose, 8.0–13.5 × 2.5–4.0 μm, with a long gradually tapering collula. ***Conidia*** spiny, fusiform to pyriform, sometimes ellipsoidal, 3.0–4.5 × 2.0–3.5 μm (av. 3.7 × 3.3 μm, n = 50). ***Sexual morph*** not observed.

##### Notes.

Currently, five species are accepted in the section Subinflati (Houbraken et al. 2020; Sun et al. 2020). *Talaromycesguiyangensis* is classified as a new lineage in the section Subinflati, forming a strongly supported clade (ML = 100%; PP = 1.0). *T.guiyangensis* is phylogenetically closely related to *T.guizhouensis*, *T.subinflatus* and *T.tzapotlensis* (Fig. 6). Morphologically, *T.guiyangensis* can be distinguished from *T.guizhouensis* by fusiform to pyriform, sometimes ellipsoidal conidia, rather than subglobose to fusiform conidia of *T.guizhouensis*; whereas the colony of *T.guiyangensis* is velvety on MEA, rather than floccose in *T.guizhouensis* (Sun et al. 2020). The conidia of *T.subinflatus* are smooth, ellipsoidal to fusiform, rather than spiny, fusiform to pyriform, sometimes ellipsoidal in *T.guiyangensis* (Yilmaz et al. 2014). Additionally, *T.subinflatus* forms ascomata, which is not seen in *T.guiyangensis* (Yilmaz et al. 2014). In addition, *T.tzapotlensis* forms smooth to finely roughened, ellipsoidal conidia, whereas *T.guiyangensis* produces spiny, fusiform to pyriform, sometimes ellipsoidal conidia (Peterson and Jurjević 2017).

#### ﻿ Talaromyces paecilomycetoides

Taxon classificationFungiEurotialesAspergillaceae

Zhi.Y. Zhang, Y.F. Han & Z.Q. Liang sp. nov.

2982753D-C91C-5978-BEBE-501BE3773C29

: 844157

Fig. 11

##### Etymology.

Refers to the production of paecilomyces-like conidiophores.

##### Type.

China: Yunnan Province, Dali City, Dali University 25°67'32"N, 100°15'70"E, soil, 2 Sept 2019, Z.Y. Zhang (HMAS 351871 holotype designated here, ex-type living culture CGMCC 3.20785 = GZUIFR 21.894); ibid., GZUIFR 21.895.

**Figure 11.**
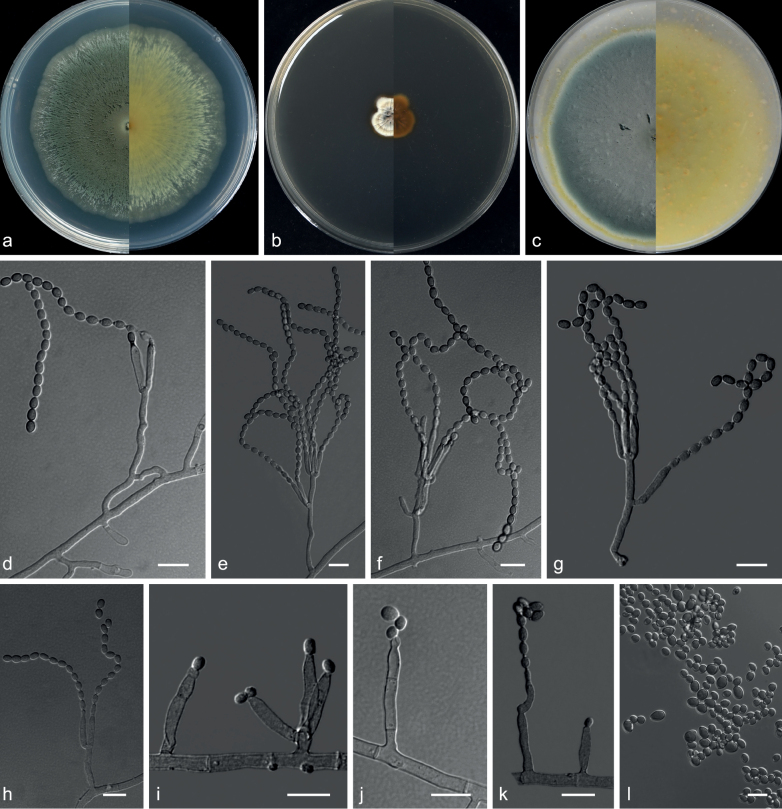
*Talaromycespaecilomycetoides* (from ex-holotype CGMCC 3.20785) **a–c** upper and reverse views of cultures on PDA, MEA and OA 14 d after inoculation **d–k** phialides and conidia **l** conidia. Scale bars: 10 µm (**d–l**).

##### Description.

***Culture characteristics*** (14 d at 25 °C): ***Colony on PDA*** 63–65 mm diam., mycelium white, planar, margins entire, slightly undulate, sporulation dense, conidia area masse dark green (28F8), soluble pigments and exudates absent; reverse greyish-green (28D5). ***Colony on MEA*** 11–13 mm diam., moderately deep, sulcate, mycelium white to buff, texture ﬂoccose, margins slightly irregular, sporulation moderately dense, conidia area masse orange white (5A2), soluble pigments and exudates absent; reverse raw umber (5F8). ***Colony on OA*** 68–70 mm diam., mycelium white, plane, texture velvety, margins entire, surrounded by an orange halo, sporulation dense, conidia area masse dark grey (1F1), soluble pigments light brown, exudates absent; reverse greyish-green (1C4).

***Hyphae*** hyaline, septate, smooth, branched, 1.0–5.0 μm wide. ***Conidiophores*** monoverticillate, smooth, irregular or absent; stipes smooth, 7–20 × 1.5–4.0 μm. ***Metulae*** 1 or absent, 10.5–14.5 × 2.0–4.0 μm. ***Phialides*** 1–4, cylindrical, flask-shaped, sometimes borne on hyphae, 10.5–20.0 × 1.5–5.0 μm. ***Conidia*** smooth, obround, ovoid, subglobose, sometimes cylindrical, 3.0–9.5 × 1.5–5.0 μm (av. 6.5 × 3.6 μm, n = 50), produced in long chains. ***Sexual morph*** not observed.

##### Additional specimens examined.

China: Yunnan Province, Kunming City, Donglu Campus of Yunnan University 25°05'51"N, 102°70'21"E, soil, 31 Aug 2019, Z.Y. Zhang, GZUIFR 21.896, ibid., GZUIFR 21.897.

##### Notes.

*Talaromycespaecilomycetoides* is one of several *Talaromyces* species with simple conidiogenous cells (Peterson and Jurjević 2017). Phylogenetically, *Talaromycespaecilomycetoides* belongs to the section Subinflati and closely related with *T.palmae* (Fig. 8). Morphologically, they can be distinguished by their conidial shape and size (obround, ovoid, subglobose, sometimes cylindrical, 3.0–9.5 × 1.5–5.0 μm in *T.paecilomycetoides*; subglobose to ellipsoidal 3–4.5 × 2–3.5 μm in *T.palmae*) (Yilmaz et al. 2014).

### ﻿Onygenales Cif. ex Benny & Kimbr.

**Arthrodermataceae Locq. ex Currah**

***Nannizzia* Stockdale**

Species of *Nannizzia* are geo- or zoophiles that occasionally infect humans (Dukik et al. 2020). With the newly-proposed taxonomy, the genus *Nannizzia* comprises 13 species (de Hoog et al. 2017; Dukik et al. 2020). However, recent work has shown that the phylogenetic relationship between *Epidermophyton* and *Nannizzia* is unstable (Baert et al. 2020).

#### ﻿ Nannizzia sinensis

Taxon classificationFungiOnygenalesArthrodermataceae

Zhi.Y. Zhang, Y.F. Han & Z.Q. Liang sp. nov.

A7FA6ACC-7A14-5117-AF7B-A3384AC46641

: 844162

Fig. 13

##### Etymology.

Refers to the country where this fungus was first isolated.

##### Type.

China: Guizhou Province, Zunyi City, the affiliated hospital of Zunyi Medical University 27°70'79"N, 106°94'54"E, soil, 5 Jul 2016, Z.Y. Zhang (HMAS 351892 holotype designated here, ex-type living culture CGMCC 3.20873 = GZUIFR 22.012).

##### Description.

***Culture characteristics*** (14 d at 25 °C): ***Colony on PDA*** 76–79 mm diam., yellowishwhite (4A2), floccose, fluffy, edge entire to diffuse; reverse white (4A1). ***Colony on MEA*** 51–52 mm diam., yellowish-white (4A2), floccose, wavy from centre to margin, edge entire; reverse white (6A1) to brownish-orange (6C8) from margin to centre. ***Colony on OA*** 52–54 mm diam., white (1A1), fluffy, sparse at the centre, margin irregular; reverse white (1A1).

***Hyphae*** hyaline, septate, smooth, branched, 1.0–4.5 μm wide; racquet hyphae and spiral hyphae not observed. ***Macroconidia*** abundant, thin- or moderately thick-walled, smooth-walled or slightly verrucose, borne individually on short branches alongside hyphae or complex branched conidiophores, fusiform, 1–5-septate, 45.0–51.0 × 11.5–12.5 μm (av. 48.6 × 12.3 μm, n = 50). ***Microconidia*** scant, sessile or short-stalked, aseptate, smooth-walled. Two types of microconidia are present: subspherical to spherical, 5.0–9.0 × 5.0–8.0 μm (av. 8.2 × 6.8 μm, n = 50); obovoidal or clavate, 3.5–6.5 × 2.0–2.5 μm (av. 5.4 × 2.3 μm, n = 50). ***Sexual morph*** not observed.

##### Additional specimens examined.

China: Hainan Province, Haikou City, Haidian Campus of Hainan University 20°05'76"N, 110°32'91"E, soil, 28 Aug 2019, Z.Y. Zhang, GZUIFR 22.054; Jiangxi Province, Jian City, Jinggangshan University, soil, 22 Aug 2019, Z.Y. Zhang, GZUIFR 22.055, ibid., GZUIFR 22.056.

##### Notes.

**Figure 12.**
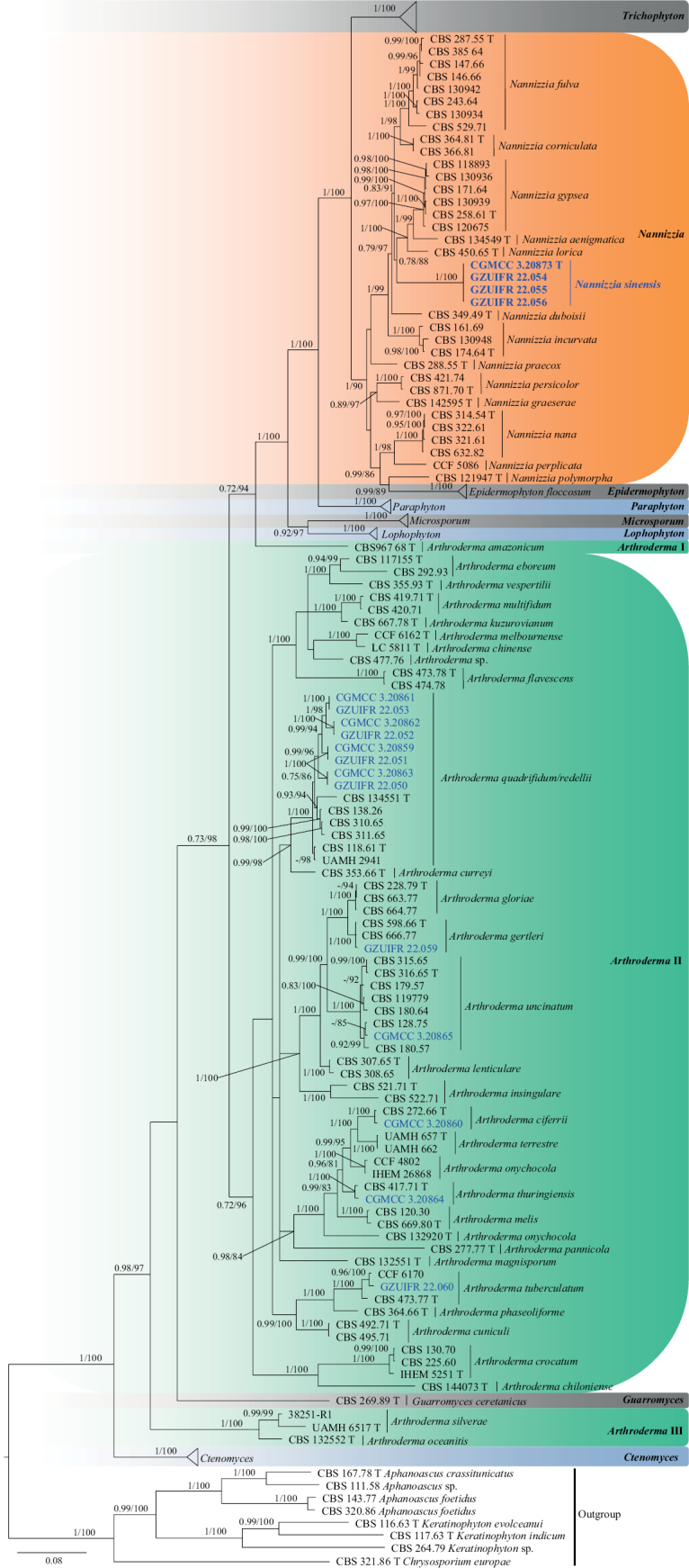
Concatenated phylogeny of the ITS, LSU, *TUB*, *TEF3* and *RP 60S L1* gene regions of species in Arthrodermataceae. Three hundred and eighteen strains are used. The tree topology of the BI was similar to the ML analysis. Bayesian posterior probability (≥ 0.7) and ML bootstrap values (≥ 70%) are indicated along branches (PP/ML). Novel species are in blue and bold font, new isolates are in blue and “T” indicates type derived sequences.

In the multi-locus phylogenetic analysis, our four isolates form a distinct clade and are closely related to *N.aenigmatica*, *N.gypsea* and *N.lorica* (Fig. 12). However, *N.sinensis* can be distinguished from *N.aenigmatica* by the presence of macroconidia (Dukik et al. 2020). In addition, *N.sinensis* differs from *N.gypsea* and *N.lorica* by the presence of subspherical to spherical microconidia (Dukik et al. 2020).

**Figure 13.**
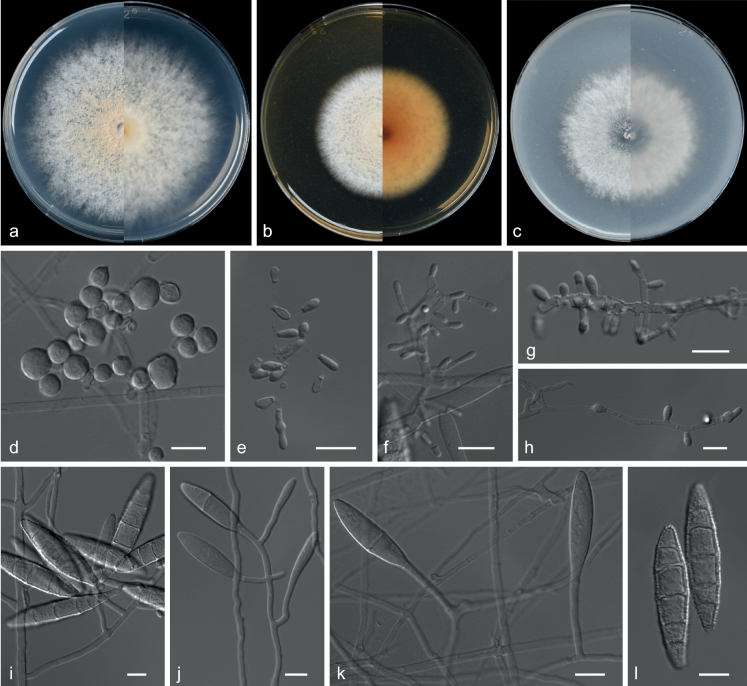
*Nannizziasinensis* (from ex-holotype CGMCC 3.20873) **a–c** upper and reverse views of cultures on PDA, MEA and OA 14 d after inoculation **d** subspherical to spherical microconidia **e–h** conidiophores with sessile or stalked microconidia **i–k** conidiophores and macroconidia **l** macroconidia. Scale bars: 10 µm (**d–l**).

### ﻿Leotiomycetes O.E. Erikss. & Winka

**Thelebolales Haeckel**

**Thelebolaceae Engl.**

***Pseudogymnoascus* Raillo**

The genus *Pseudogymnoascus* was erected by Raillo (1929). However, Raillo did not formally specify a type strain for the genus. Many years later, Samson (1972) designated *P.roseus* Raillo CBS 395.65 as the neotype. Currently, the genus *Pseudogymnoascus* consists of 18 valid species (Villanueva et al. 2021; Zhang et al. 2021b), which belong to 13 clades (Minnis and Lindner 2013).

#### ﻿ Pseudogymnoascus botryoides

Taxon classificationFungiThelebolalesThelebolaceae

Zhi.Y. Zhang, Y.F. Han & Z.Q. Liang sp. nov.

B1865303-5FD9-54E3-B4A3-A21D2B9A810E

: 844164

Fig. 15

##### Etymology.

In reference to aleurioconidia and intercalary conidia which are borne together to form a botryoidal-like structure.

##### Type.

China: Guangxi Zhuang Autonomous Region, Beihai City 21°48'75"N, 109°12'72"E, soil, 10 Jul 2016, Z.Y. Zhang (HMAS 351904 holotype designated here, ex-type living culture CGMCC 3.20875 = GZUIFR 22.024); ibid., GZUIFR 22.044.

##### Description.

***Culture characteristics*** (14 d at 25 °C): ***Colony on PDA*** 29–30 mm diam., annular, margin aerial hyphae sparse, orange white (5A2) to white (5A1) from centre to margin, flat, compact, exudates and diffusible pigments absent; reverse reddish-orange (7A8) to orange white (5A2) from centre to margin. ***Colony on MEA*** 28–29 mm diam., white (7A1), flat, compact, nearly round, margin regular, exudates abundant, light red, diffusible pigments absent, reverse brown (7E8) to white (7A1) from centre to margin. ***Colony on OA*** greyish-orange (5B3) to white (5A1) from centre to margin, 25–28 mm diam., flocculent, granuliform, nearly round, margin slightly undulated, exudates abundant, diffusible pigments absent; reverse light orange (5A5) to white (5A1) from centre to margin.

***Hyphae*** branched, septate, hyaline, smooth, 0.5–2.5 μm diam. wide, fertile hyphae bearing aleurioconidia and/or intercalary conidia, sessile. Aleurioconidia and intercalary conidia are borne together to form a botryoidal-like structure. ***Conidiophores*** abundant, dense, interwoven into a network, curved, hyaline, rough, usually bearing verticils of two to eight branches, arising from the stipe at an acute angle. ***Aleurioconidia*** pyriform, obovoid, elongated, with a broad truncated basal scar, 2.0–4.5 × 1.5–2.5 µm (av. 3.8 × 2.3 μm, n = 50). ***Intercalary conidia*** drum, reniform, 2.5–5.0 × 1.5–2.5 µm, separated by connective cells that undergo rhexolysis, bearing sessile conidia. ***Arthroconidia*** rare, cylindrical, sometimes slightly curved, 2.5–5.0 × 1.0–2.0 µm (av. 4.4 × 1.6 μm, n = 50). ***Sexual morph*** unknown.

##### Additional specimens examined.

China: Guangdong Province, Zhanjiang City, the affiliated hospital of Guangdong Medical University 21°19'98"N, 110°40'34"E, soil, 25 Aug 2019, Z.Y. Zhang, GZUIFR 22.045, ibid., GZUIFR 22.046.

##### Notes.

**Figure 14.**
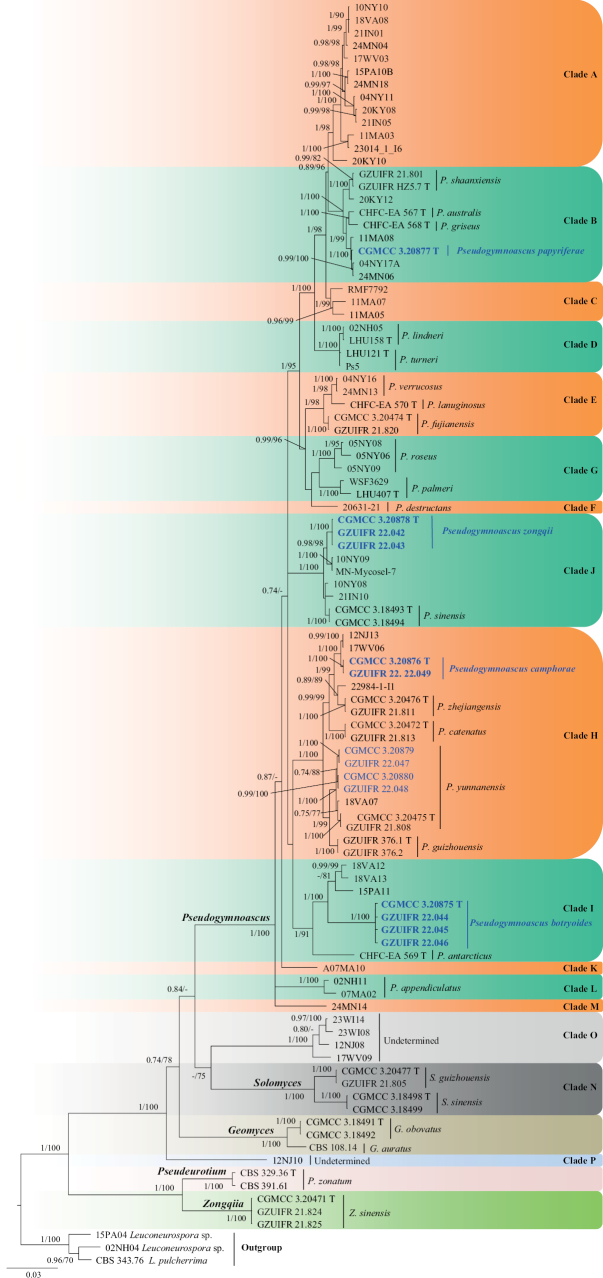
Concatenated phylogeny of the ITS, LSU, *EF1A*, *RPB2* and *MCM7* gene regions of species in Thelebolaceae. Ninety-nine strains are used. The tree is rooted in *Leuconeurosporapulcherrima* (CBS 343.76) and *Leuconeurospora* sp. (15PA04 and 02NH04). The tree topology of the BI was similar to the ML analysis. Bayesian posterior probability (≥ 0.7) and ML bootstrap values (≥ 70%) are indicated along branches (PP/ML). Novel species are in blue and bold font, new isolates are in blue and “T” indicates type derived sequences.

*Pseudogymnoascusbotryoides* was placed as a member of clade I (Fig. 14). Clade I is composed of *P.antarcticus* and many other isolates that remain unidentified species (Minnis and Lindner 2013; Villanueva et al. 2021). Phylogenetically, *P.botryoides* forms a distinct lineage with strong support (Fig. 14). Morphologically, *P.botryoides* can be distinguished from other species in the genus *Pseudogymnoascus* by its aleurioconidia and intercalary conidia being borne together to form a botryoidal-like structure.

**Figure 15.**
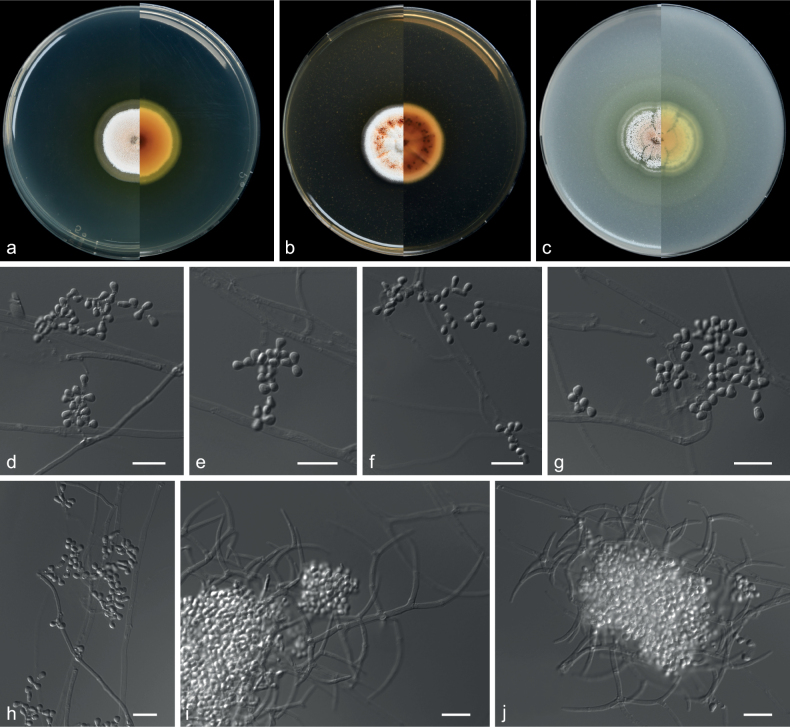
*Pseudogymnoascusbotryoides* (from ex-holotype CGMCC 3.20875) **a–c** upper and reverse views of cultures on PDA, MEA and OA 14 d after inoculation **d–h** conidiophores and conidia **i, j** cluster conidia. Scale bars: 10 µm (**d–j**).

#### ﻿ Pseudogymnoascus camphorae

Taxon classificationFungiThelebolalesThelebolaceae

Zhi.Y. Zhang, Y.F. Han & Z.Q. Liang sp. nov.

E6489EB1-0E1C-5458-9E70-6840900CFB02

: 844165

Fig. 16

##### Etymology.

Referring to the type strains first isolated from epiphytic soil of *Cinnamomumcamphora* (Linn) Presl.

##### Type.

China: Guizhou Province, Guiyang City, South Campus of Guizhou University 26°42'21"N, 106°67'13"E, epiphytic soil of *C.camphora*, 8 Jul 2018, Z.Y. Zhang (HMAS 351901 holotype designated here, ex-type living culture CGMCC 3.20876 = GZUIFR 22.021).

##### Description.

***Culture characteristics*** (14 d at 25 °C): ***Colony on PDA*** 20–22 mm diam., white (6A1), slightly raised, flocculent, margin irregular, localised bulge, exudates and diffusible pigments absent; reverse dark brown (6F8) to light brown (6D4) from centre to margin. ***Colony on MEA*** 20–21 mm diam., white (5A1) to yellowish-white (4A2) from centre to margin, flocculent, nearly round, margin undulated, exudates and diffusible pigments absent; reverse yellowish-brown (5E8) to orange (5A7) from centre to margin. ***Colony on OA*** 19–20 mm diam., white (13A1), slightly raised, fluffy, nearly round, margin regular, exudates absent, diffusible pigments abundant, pewter; reverse reddish (13A2).

**Figure 16.**
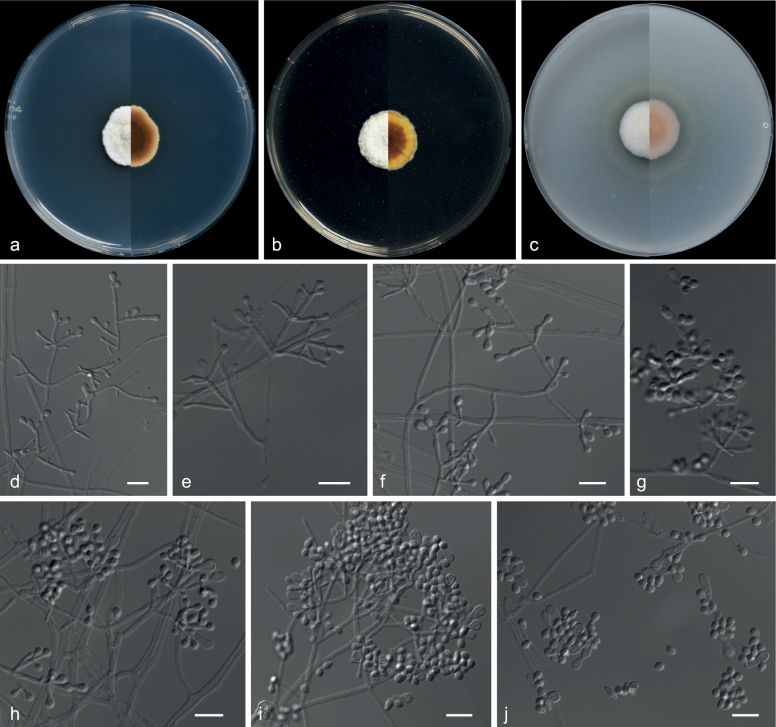
*Pseudogymnoascuscamphorae* (from ex-holotype CGMCC 3.20876) **a–c** upper and reverse views of cultures on PDA, MEA and OA 14 d after inoculation **d–i** conidiophores and conidia **j** conidia. Scale bars: 10 µm (**d–j**).

***Hyphae*** branched, septate, hyaline, smooth, 1.0–3.0 μm diam. ***Conidiophores*** abundant, solitary, sometimes minimally differentiated from hyphae, hyaline, smooth, arising from erect hyphae, usually bearing verticils of two to four branches at an acute angle. ***Aleurioconidia*** pyriform, obovoid, with a broad truncated basal scar, 4.0–5.5 × 3.0–3.5 µm (av. 5.2 × 3.2, n = 50). ***Terminal aleurioconidia*** at the axis obovoid, clavate or irregular, solitary or two in chains, 6.0–10.0 × 3.0–3.5 µm (av. 8.7 × 3.2 μm, n = 50), with a broad truncated basal scar. Intercalary conidia subglobose, drum, obovoid, 3.0–4.0 × 2.0–3.0 µm (av. 3.5 × 2.6 μm, n = 50), sometimes separated by connective cells that undergo rhexolysis. ***Arthroconidia*** absent. ***Sexual morph*** unknown.

##### Additional specimens examined.

China: Fujian Province, Xiamen City, Wuyuanbay Wetland Park 24°51'52"N, 118°17'48"E, soil, 19 Aug 2019, Z.Y. Zhang, GZUIFR 22. 22.049.

##### Notes.

*Pseudogymnoascuscamphorae* was placed as a member of clade H (Fig. 14). *P.camphorae* is phylogenetically related to *P.zhejiangensis* and *P.catenatus*; however, *P.camphorae* still forms a single clade. Morphologically, *P.camphorae* can be differentiated from *P.catenatus* by its absent arthroconidia (Zhang et al. 2021b). *P.camphorae* is distinguished from *P.zhejiangensis* by the size and shape of its aleurioconidia (pyriform, obovoid, 4.0–5.5 × 3.0–3.5 µm vs. obovoid to globose, 2.5–4.5 × 2.5–4.0 µm, respectively) and terminal aleurioconidia (obovoid, clavate or irregular, solitary or two in chains, 6.0–10.0 × 3.0–3.5 µm vs. clavate, long obovoid, 5–9 × 2.5–4 µm, respectively) (Zhang et al. 2021b).

#### ﻿ Pseudogymnoascus papyriferae

Taxon classificationFungiThelebolalesThelebolaceae

Zhi.Y. Zhang, Y.F. Han & Z.Q. Liang sp. nov.

697A88D3-079D-587A-9FDA-D5A09FD0C085

: 844166

Fig. 17

##### Etymology.

Referring to the type strain isolated from epiphytic soil of *Broussonetiapapyrifera* L’Heritier ex Ventenat.

##### Type.

China: Shaanxi Province, Hanzhong City 33°07'65"N, 107°03'13"E, from epiphytic soil of *B.papyrifera*, Sep 2018, Z.Y. Zhang (HMAS 351878 holotype designated here, ex-type living culture CGMCC 3.20877 = GZUIFR 22.020).

##### Description.

***Culture characteristics*** (14 d at 25 °C): ***Colony on PDA*** 13–15 mm diam., light orange (6A4) to white (6A1) from centre to margin, slightly raised, cottony, floccose, nearly round, margin slightly undulated, abundant exudates in the form of transparent, cinnamon-colour droplets of large size, diffusible pigments absent; reverse rust brown (6E8) to white (6A1). ***Colony on MEA*** 13–14 mm diam., white (2A1), hyphae kink into bundles, slightly raised in the centre, nearly round, exudates and diffusible pigments absent; reverse white (2A1) to yellowish-white (2A2) from margin to centre. ***Colony on OA*** 14–15 mm diam., white (1A1), powdery with dense in the middle with sparse margins, slightly raised at the centre, nearly round, margin slightly undulate, exudates absent, producing a diffusible faint white pigment; reverse white (1A1).

**Figure 17.**
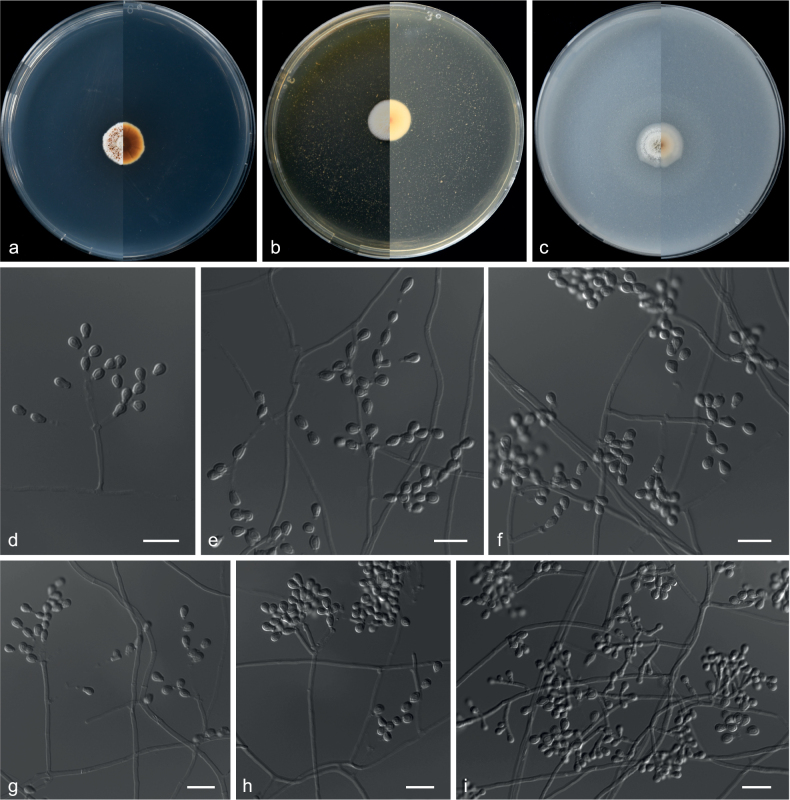
*Pseudogymnoascuspapyriferae* (from ex-holotype CGMCC 3.20877) **a–c** upper and reverse views of cultures on PDA, MEA and OA 14 d after inoculation **d–i** conidiophore and conidia. Scale bars: 10 µm (**d–i**).

***Hyphae*** branched, septate, hyaline, smooth, 1.0–2.5 μm diam. Sometimes lateral hyphae end in chains of a barrel- or fusiform shape with blunt-ended arthroconidia, sometimes bearing aleurioconidia, sessile or stalked. ***Conidiophores*** abundant, solitary, erect, arising in acute angles with the main axis, hyaline, smooth, usually bearing verticils of two to four branches arising from the stipe at an acute angle. ***Aleurioconidia*** obovoid, pyriform to subglobose, with a broad truncated basal scar, 3.5–6.0 × 2.5–4.0 µm (av. 4.4 × 3.5 μm, n = 50), in conidiophores separated by connective cells. ***Intercalary conidia*** drum-shaped, barrel-shaped, pyriform to elongated, with a broad truncated scar at the basal or both ends, 3.5–5.5 × 2.5–3.5 µm (av. 5.2 × 3.4 μm, n = 50). ***Arthroconidia*** rare, cylindrical to slightly inflated in the middle, 2.5–4.5 × 2.0–2.5 µm (av. 3.4 × 2.2 μm, n = 50). Arthroconidia chain or separated by connective cells that undergo rhexolysis, occasionally bearing sessile conidia. ***Sexual morph*** unknown.

##### Notes.

*Pseudogymnoascuspapyriferae* is nested in clade B (Fig. 14). Clade B is composed of three species (*P.shaanxiensis*, *P.australis* and *P.griseus*) and six other strains that remain unidentiﬁed species (Minnis and Lindner 2013; Zhang et al. 2020b, 2021b; Villanueva et al. 2021). Phylogenetic analysis clearly shows that *P.papyriferae* forms a distinct lineage (Fig. 14). *Pseudogymnoascuspapyriferae* can be distinguished from *P.shaanxiensis* by the presence of arthroconidia (Zhang et al. 2020b). *Pseudogymnoascuspapyriferae* can be differentiated from *P.australis* by the shape of intercalary conidia (drum-shaped, barrel-shaped, pyriform to elongated vs. subglobose to elongated and barrel-shaped, respectively) and rarely arthroconidia (Villanueva et al. 2021). In addition, *P.papyriferae* differs from *P.griseus* in the size and shape of its intercalary conidia (3.5–5.5 × 2.5–3.5 µm, drum-shaped, barrel-shaped, pyriform to elongated vs. 3.5–9.6 × 1.7–3.9 µm, subglobose to elongated and barrel-shaped, respectively) (Villanueva et al. 2021).

#### ﻿ Pseudogymnoascus zongqii

Taxon classificationFungiThelebolalesThelebolaceae

Zhi.Y. Zhang, Y.F. Han & Z.Q. Liang sp. nov.

0D8293C8-5C93-542E-BA31-43C51B60C42B

: 844168

Fig. 18

##### Etymology.

Refers to the name of Prof. Zong-Qi Liang.

##### Type.

China: Sichuan Province, Chengdu City 30°65'96"N, 104°04'44"E, soil, 8 Aug 2016, Z.Y. Zhang (HMAS 351905 holotype designated here, ex-type living culture CGMCC 3.20878 = GZUIFR 22.025).

##### Description.

***Culture characteristics*** (14 d at 25 °C): ***Colony on PDA*** 13–15 mm diam., pale orange (6A3) to white (6A1) from centre to margin, fluffy, flocculent, nearly round, margin slightly sunken, exudates absent, diffusible pigments transparent and inconspicuous; reverse brownish-grey (6C2) to light orange (6A5) from centre to margin. ***Colony on MEA*** 12–13 mm diam., light yellow (4A4) to white (4A1) from centre to margin, hyphae kink into bundles, raised at the centre, nearly round, margin regular, exudates and diffusible pigments absent; reverse chrome yellow (5B8) to light yellow (4A4) from centre to margin. ***Colony on OA*** 12 mm diam., grey (5C1) to white (5A1) from centre to margin, flocculent, dense at the centre, sparse at margins, nearly round, margin regular, exudates absent, diffusible pigments transparent and inconspicuous; reverse raw umber (5F8) to grey (5D1) from centre to margin.

**Figure 18.**
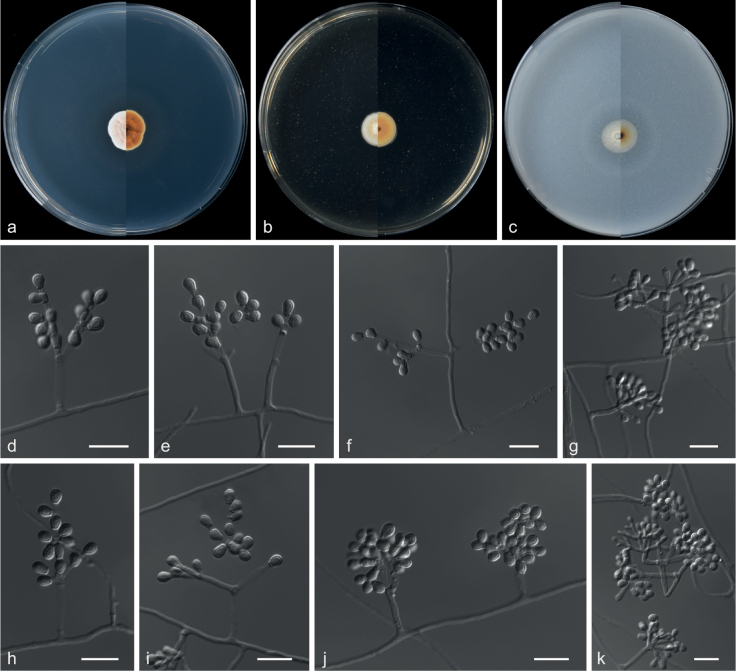
*Pseudogymnoascuszongqii* (from ex-holotype CGMCC 3.20878) **a–c** upper and reverse views of cultures on PDA, MEA and OA 14 d after inoculation **d–f, h, i** conidia and intercalary conidia **g–k** conidiophore and conidia. Scale bars: 10 µm (**d–k**).

***Hyphae*** branched, septate, hyaline, smooth, 1.0–3.0 μm diam. wide. ***Conidiophores*** abundant, solitary, sometimes minimally differentiated from hyphae, hyaline, smooth, arising from the erect hyphae, usually bearing verticils of two to five branches at an acute angle. Aleurioconidia and intercalary conidia are abundant, hyaline, smooth or rough. ***Aleurioconidia*** pyriform, occasionally obovoid to subglobose, with a broad truncated basal scar, 3.0–5.0 × 2.5–3.5 µm (av. 4.3 × 3.2 μm, n = 50). ***Intercalary conidia*** pyriform to obovoid, 3.5–5.0 × 2.5–4.5 µm (av. 4.6 × 3.8 μm, n = 50), separated by connective cells that undergo rhexolysis; occasionally bearing sessile conidia. ***Arthroconidia*** absent. ***Sexual morph*** unknown.

##### Additional specimens examined.

China: Guizhou Province, Zunyi City, the affiliated hospital of Zunyi Medical University 27°70'79"N, 106°94'54"E, soil, 11 Sept 2016, Z.Y. Zhang, GZUIFR 22.042, ibid., GZUIFR 22.043.

##### Notes.

*Pseudogymnoascuszongqii* was placed as a member of clade J (Fig. 14). Clade J is composed of *P.sinensis* and many other strains that remain unidentified species (Minnis and Lindner 2013; Zhang et al. 2020b). Phylogenetically, *P.zongqii* forms a distinct lineage with strong support (Fig. 14). Morphologically, *P.zongqii* can be distinguished from *P.sinensis* by its subglobose conidia and absence of drum- or irregularly shaped intercalary conidia (Zhang et al. 2020b).

### ﻿Sordariomycetes O.E. Erikss. & Winka

**Hypocreales Lindau**

**Bionectriaceae Samuels & Rossman**

***Clonostachys* Corda**

Corda (1839) introduced the genus *Clonostachys*, based on *C.araucaria*. *Clonostachys* species are characterised by penicillate, sporodochial or dimorphic conidiophores and phialidic conidiogenous cells, producing hyaline conidia (Schroers 2001). Rossman et al. (2013) linked the sexual morphic genus *Bionectria* with *Clonostachys* and *Bionectria* was synonymised under *Clonostachys*. *Clonostachys* is a species-rich genus with more than 107 records listed in the Index Fungorum (http://www.indexfungorum.org, accessed on 14 May 2022). Members of the genus *Clonostachys* are saprotrophs and are heavily parasitic on fungi and lichens or inhabit recently dead trees and decaying leaves (Schroers 2001). However, *Clonostachys* spp. are rarely found to be parasitic on myxomycetes, insects, nematodes, flatworms, molluscs or oomycetes (Schroers 2001).

#### ﻿ Clonostachys shanghaiensis

Taxon classificationFungiHypocrealesBionectriaceae

Zhi.Y. Zhang, Y.F. Han & Z.Q. Liang sp. nov.

AF987E1E-AFB0-56A0-B776-4820B526762A

: 844171

Fig. 20

##### Etymology.

In reference to Shanghai, the city where the type specimen was obtained.

##### Type.

China: Shanghai Municipality, Shanghai People’s Park 31°23'33"N, 121°47'29"E, soil, 15 Aug 2020, Z.Y. Zhang (HMAS 351878 holotype designated here, ex-type living culture CGMCC 3.20773 = GZUIFR 21.915).

##### Description.

***Culture characteristics*** (14 d at 25 °C): ***Colony on PDA*** 50 mm diam., white (9A1), flat, cottony, annular, dense at the centre, margin slightly undulated; reverse reddish-white (9A2). ***Colony on MEA*** 66 mm diam., white (5A1), flat, margin undulated, white; reverse orange white (5A2). ***Colony on OA*** 63 mm diam., white (9A1), surface undulated, margin entire; reverse reddish-white (9A2).

***Hyphae*** branched, septate, hyaline, smooth, 1.0–5.0 μm diam. ***Conidiophores*** arising from aerial hyphae, monomorphic, hyaline, smooth-walled, solitary, not sporodochial, monoverticillate or biverticillate, 3.5–9.0 × 1.5–3.0 µm. ***Phialides*** solitary or in whorls 2–5, broadly flask-shaped, slightly tapering towards the apex, with visible periclinal thickening, hyaline, smooth-walled, borne on the tip of hyphae or conidiophores, 2.5–10.0 × 2.0–3.5 µm. ***Intercalary phialides*** are rarely observed. ***Conidia*** hyaline, smooth, ellipsoidal, oblong to olivary, from phialides, 3.0–5.5 × 2.0–3.0 μm (av. 4.8 × 2.5 μm, n = 50). ***Sexual morph*** not observed.

##### Additional specimens examined.

China: Shanghai Municipality, South Campus of Fudan University 31°29'30"N, 121°50'03"E, soil, 16 Aug 2020, Z.Y. Zhang, GZUIFR 21.916.

##### Notes.

**Figure 19.**
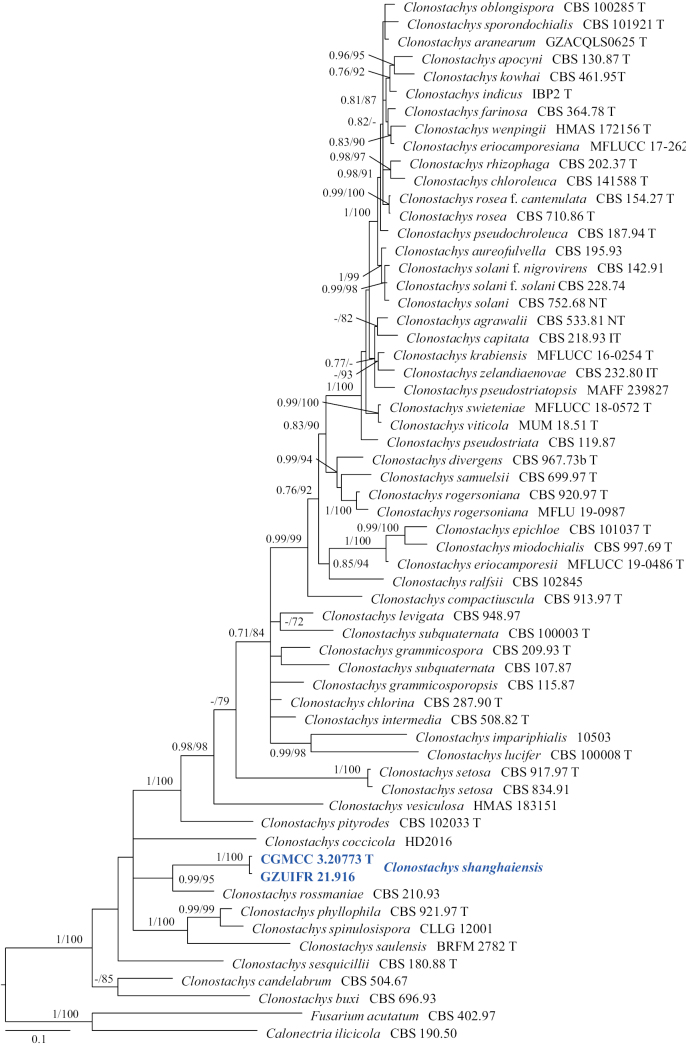
Concatenated phylogeny of the ITS and *TUB* gene regions of species in *Clonostachys*. Sixty strains are used. The tree is rooted in *Fusariumacutatum* (CBS 402.97) and *Calonectriailicicola* (CBS 190.50). The tree topology of the BI was similar to the ML analysis. Bayesian posterior probability (≥ 0.7) and ML bootstrap values (≥ 70%) are indicated along branches (PP/ML). Novel species are in blue and bold font and “T” indicates type derived sequences.

Our new isolates (CGMCC 3.20773 and GZUIFR 21.916) formed a single clade and are closely related to *Clonostachysrossmaniae* (CBS 210.93) (Fig. 19). However, the conidia of *C.shanghaiensis* are ellipsoidal, oblong to olivary, rather than ellipsoidal to ovoidal in *C.rossmaniae* (Schroers 2001). Therefore, this species is regarded as a new species, based on morphology and multi-locus phylogeny.

**Figure 20.**
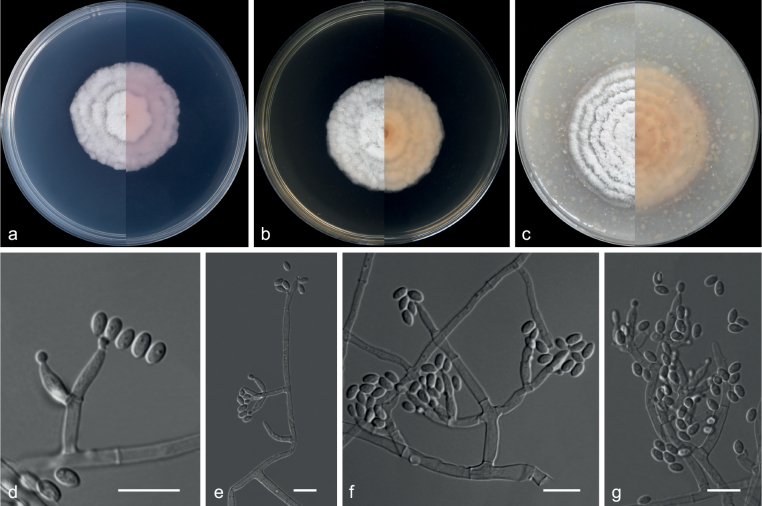
*Clonostachysshanghaiensis* (from ex-holotype CGMCC 3.20773) **a–c** upper and reverse views of cultures on PDA, MEA and OA 14 d after inoculation **d–g** conidiophore and conidia. Scale bars: 10 µm (**d–g**).

### ﻿Nectriaceae Tul. & C. Tul.

***Cyanonectria* Samuels & P. Chaverri**

Samuels et al. (2009) established the genus *Cyanonectria*, with the type species *Cyanonectriacyanostoma*. Currently, this genus includes two accepted species (Crous et al. 2021), both are isolated from branches of *Buxussempervirens* L. (Schroers et al. 2011; Crous et al. 2021).

#### ﻿ Cyanonectria bispora

Taxon classificationFungiHypocrealesNectriaceae

Zhi.Y. Zhang, Y.F. Han & Z.Q. Liang sp. nov.

72FBB5CE-21D1-5D94-9452-8CC2DFB7F33C

: 844172

Fig. 22

##### Etymology.

In reference to its production of both macroconidia and microconidia.

##### Type.

China: Yunnan Province, Dali City, Dali Bai Autonomous Prefecture People’s Hospital 25°57'89"N, 100°22'16"E, soil, 3 Sep 2019, Z.Y. Zhang (HMAS 351875 holotype designated here, ex-type living culture CGMCC 3.20774 = GZUIFR 21.908).

##### Description.

***Culture characteristics*** (14 d at 25 °C): ***Colony on PDA*** 42–46 mm diam., grey (29B1), flocculent, aerial mycelium sparse, substrate mycelium abundant, fimbriate; reverse grey (29C1). ***Colony on MEA*** 15–19 mm diam., grey (29B1) to white (29A1) from centre to margin, fluffy, margin dentate; reverse grey (29F1) to white (29A1) from centre to margin. ***Colony on OA*** 63 mm diam., yellowish-white (4A2), felty, rounded, margin regular; reverse pale yellow (4A3).

***Hyphae*** branched, septate, hyaline, smooth, 1.0–5.0 μm diam. ***Conidiophores*** mononematous (aerial conidiophores) or grouped on sporodochia. ***Monophialides*** arising from aerial hyphae, hyaline, smooth-walled, solitary or connected by pronounced connectors, sometimes septate, cylindrical, sometimes curved irregularly, neck broadly tapering towards the apex, 5.5–39.0 μm long, 1.0–3.5 μm wide at the base, ca. 1.0–2.5 μm near the aperture. ***Sporodochia*** of branched conidiophores with solitary or whorls of 2–3 terminal monophialides. ***Phialides*** of sporodochia cylindrical or bottle-shaped, 13.5–29 μm long, 2.0–3.5 μm wide at the base, 2.5–4.0 μm in middle, 1.0–2.0 μm wide near the conidiogenous aperture. ***Microconidia*** hyaline, smooth, aseptate, cylindrical, fusiform, sometimes irregular, 5.0–14.5 × 2.5–6.0 μm (av. 10.4 × 4.6 μm, n = 50). ***Macroconidia*** hyaline, smooth, typically with the central and basal part nearly straight, rarely gently curved throughout, with a more or less pronounced pedicellate foot cell and an inequilateral fusoid or hooked apical cell, aseptate or 1–3(–4) septate, 23.5–40.5 × 3.0–5.5 μm (av. 38.6 × 4.8 μm, n = 50). ***Chlamydospores*** not observed. ***Sexual morph*** not observed.

##### Additional specimens examined.

China: Guangxi Zhuang Autonomous Region, Guilin City, Yucai Campus of Guangxi Normal University 25°26'64"N, 110°32'70"E, soil, 30 Aug 2019, Z.Y. Zhang, GZUIFR 21.909.

##### Notes.

**Figure 21.**
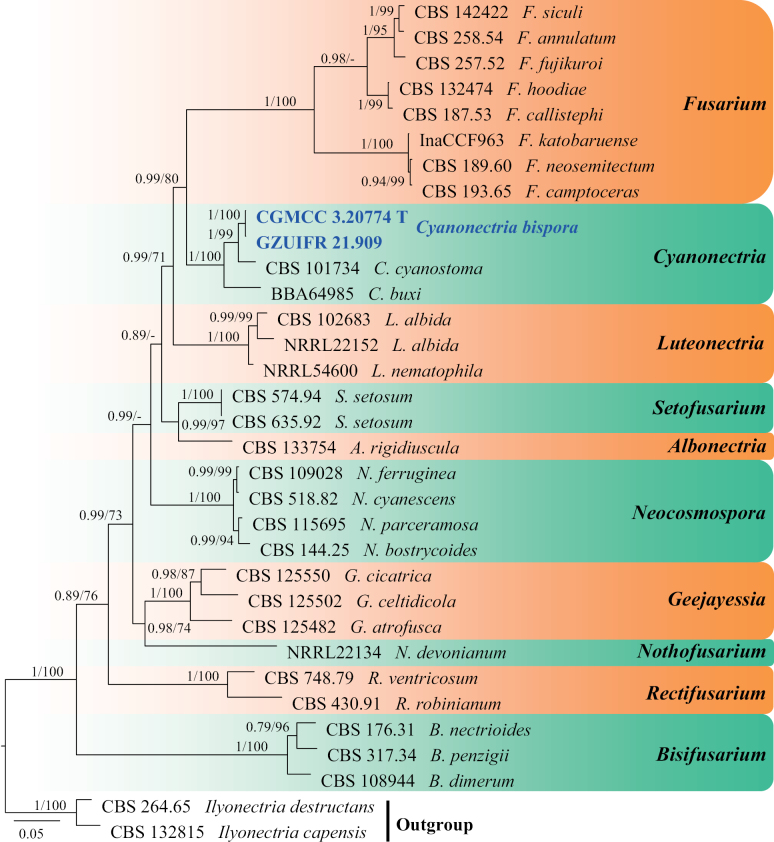
Concatenated phylogeny of the ITS, LSU, *RPB2* and *EF1A* gene regions of species in *Cyanonectria* and its allied genera. Thirty-three strains are used. The tree is rooted in *Ilyonectriadestructans* (CBS 264.65) and *I.capensis* (CBS 132815). The tree topology of the BI was similar to the ML analysis. Bayesian posterior probability (≥ 0.7) and ML bootstrap values (≥ 70%) are indicated along branches (PP/ML). Novel species are in blue and bold font and “T” indicates type derived sequences.

The species is described here, based on its morphology of the asexual morph. In the phylogenetic analysis (Fig. 21), *Cyanonectriabispora* is nested in the genus *Cyanonectria* and sister to *C.cyanostoma* and *C.buxi*. Morphologically, *C.bispora* can be distinguished from *C.cyanostoma* and *C.buxi* by the production of macroconidia and microconidia (Samuels et al. 2009). In addition, *C.cyanostoma* and *C.buxi* are reported from Europe (Belgium, France, Germany and Slovenia) and associated with *B.sempervirens*, while *C.bispora* was isolated from soil in China.

**Figure 22.**
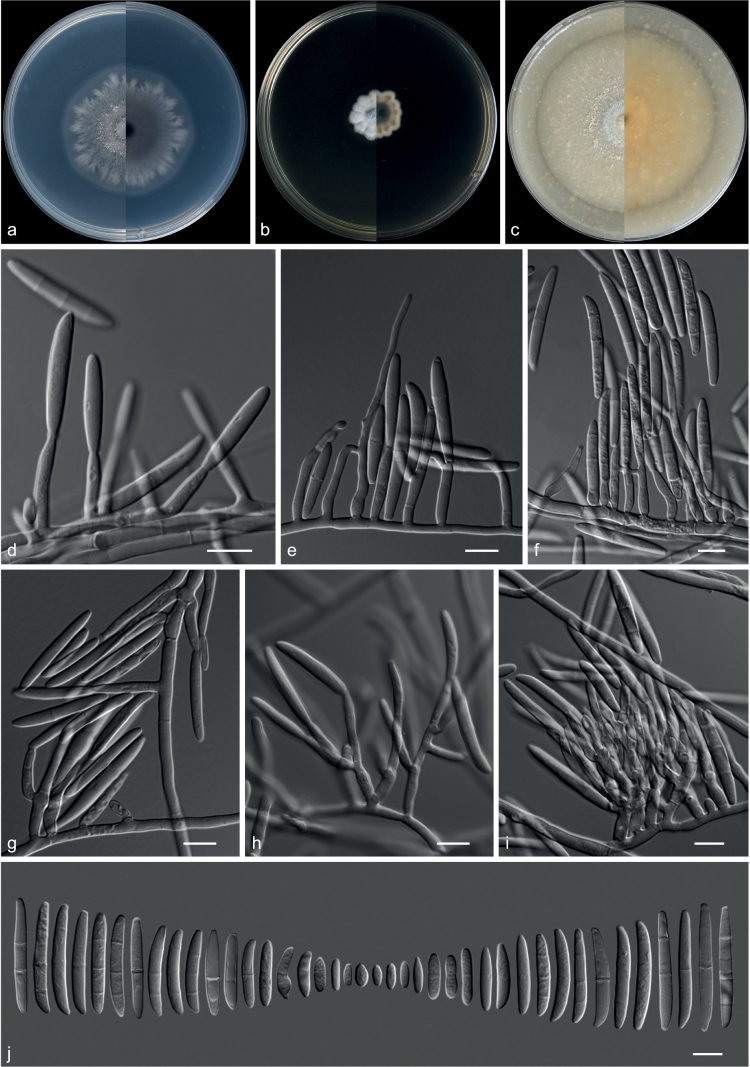
*Cyanonectriabispora* (from ex-holotype CGMCC 3.20774) **a–c** upper and reverse views of cultures on PDA, MEA and OA 14 d after inoculation **d–f** monophialide and conidia **g, h** polyphialides with multiple conidiogenous loci **i** sporodochial conidiophore and conidiogenous cells **j** conidia. Scale bars: 10 µm (**d–j**).

### ﻿*Fusarium* Link

The genus *Fusarium* was established in 1809 by Link. Currently, *Fusarium* consists of 18 species complexes (Sandoval-Denis et al. 2018a; Lombard et al. 2019; Crous et al. 2021) with over 100 species. While the current classification of *Fusarium* relies on molecular phylogeny, it is important to note that morphology remains an essential component of the definition of fungal genus and species and should not be disregarded (Crous et al. 2021).

#### ﻿ Fusarium brachypodum

Taxon classificationFungiHypocrealesNectriaceae

Zhi.Y. Zhang, Y.F. Han & Z.Q. Liang sp. nov.

12A87D33-0ABC-5EB0-92DA-FDED956E965F

: 844173

Fig. 24

##### Etymology.

Refers to the sporodochial conidia connected by 1–3 short-stalks.

##### Type.

China: Guizhou Province, Guiyang City, Qianlingshan Park 26°59'03"N, 106°69'57"E, soil, 13 Sep 2019, Z.Y. Zhang (HMAS 351876 holotype designated here, ex-type living culture CGMCC 3.20776 = GZUIFR 21.910).

##### Description.

***Culture characteristics*** (14 d at 25 °C): ***Colony on PDA*** 79 mm diam., milk white (1A2), flat, velvety, with scant and short aerial mycelium, rounded, margins irregular; reverse white (1A1). ***Colony on MEA*** 63 mm diam., white (1A1), flat, cottony, rounded, margins regularly; reverse white (1A1). ***Colony on OA*** 58 mm diam., white (1A1), flat, surface khaki granular, rounded, margin entire; reverse white (1A1).

***Hyphae*** abundant, branched, septate, hyaline, smooth, 1.0–4.0 μm diam. ***Conidiophores*** arising from aerial hyphae, straight or flexuous, hyaline, smooth-walled, unbranched or sparingly branched, bearing terminal or monophialides, often reduced to single phialides. ***Phialides*** subcylindrical to cylindrical, straight or flexuous, smooth, 12–22 μm long, 3.5–4.0 μm at the widest point. ***Aerial conidia*** forming small false heads on the tips of the phialides, hyaline, subcylindrical to cylindrical, straight or flexuous, smooth-walled, aseptate, 11.5–34.0 × 2.5–4.5 µm (av. 24.5 × 3.6 μm, n = 50). ***Sporodochia*** abundant. ***Conidiophores*** in sporodochia verticillately branched, consisting of a short, smooth-walled stipe, phialides cylindrical to lageniform, or irregular, 12.5–18.5 × 2.5–4.5 µm, bearing apical whorls of 2–3 monophialides or as single lateral monophialide. ***Sporodochial conidia*** smooth-walled, lunate to falcate, curved or somewhat straight, robust, with an elongated or whip-like curved apical cell and papillate to elongate, well-developed foot-shaped, sometimes poorly development basal cell, always aggregated, with connected by 1–3 short-stalked; 1–3 septate, sometimes aseptate, 1–septate conidia: 26.5–29 × 3.5–6.0 μm (av. 28.2 × 4.6 μm, n = 50), 2–septate conidia: 38.0–45.0 × 4.5–6.0 μm (av. 41.7 × 5.5 μm, n = 50), 3–septate conidia: 32.5–61.0 × 4.0–6.0 μm (av. 46.0 × 4.3 μm, n = 50), aseptate conidia: 24.5–45.0 × 3.5–6.0 μm (av. 34.7 × 4.0 μm, n = 50). ***Chlamydospores*** rare, subglobose to globose, hyaline, smooth-walled, intercalary, solitary, 9.0–13.5 μm (av. 11.8 μm, n = 5) diam. ***Coiled*** sometimes from the substrate and aerial mycelium. ***Microconidia*** not observed. ***Sexual morph*** unknown.

##### Additional specimens examined.

China: Jiangxi Province, Jian City, Jinggangshan University 27°11'30"N, 115°03'19"E, soil, 22 Aug 2019, Z.Y. Zhang, GZUIFR 21.911.

##### Notes.

**Figure 23.**
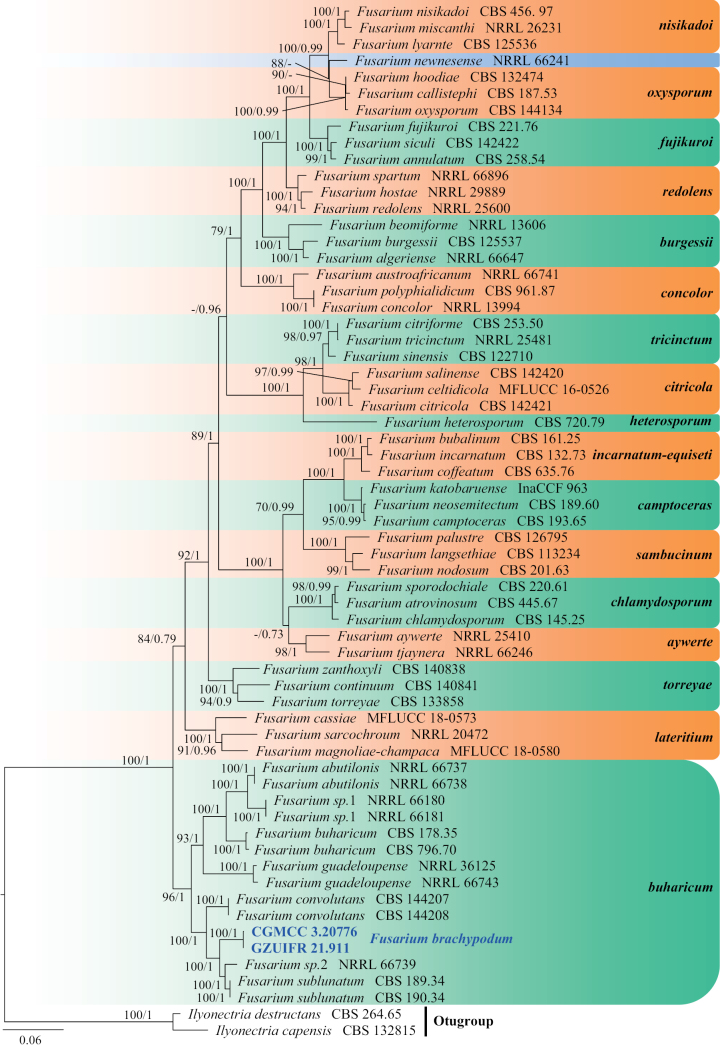
Concatenated phylogeny of the ITS, LSU, *RPB2* and *EF1A* gene regions of species in *Fusarium*. Sixty-three strains are used. The tree is rooted in *Ilyonectriadestructans* (CBS 264.65) and *I.capensis* (CBS 132815). The tree topology of the BI was similar to the ML analysis. Bayesian posterior probability (≥ 0.7) and ML bootstrap values (≥ 70%) are indicated along branches (PP/ML). Novel species are in blue and bold font and “T” indicates type derived sequences.

*Fusariumbrachypodum* was introduced as a new species while adding one more species to the *Fusariumbuharicum* species complex (FBSC; Geiser et al. 2013; O’Donnell et al. 2013). Our isolates formed a single clade nested in FBSC (Fig. 23), which comprise *F.abutilonis*, *F.buharicum*, *F.convolutans*, *F.guadeloupense* and *F.sublunatum*. However, *Fusariumbrachypodum* differs from members of FBSC in its presence of hyphae coiled, chlamydospores, absent microconidia and sporodochial conidia connected by 1–3 short stalks (Gerlach and Nirenberg 1982; Sandoval-Denis et al. 2018b).

**Figure 24.**
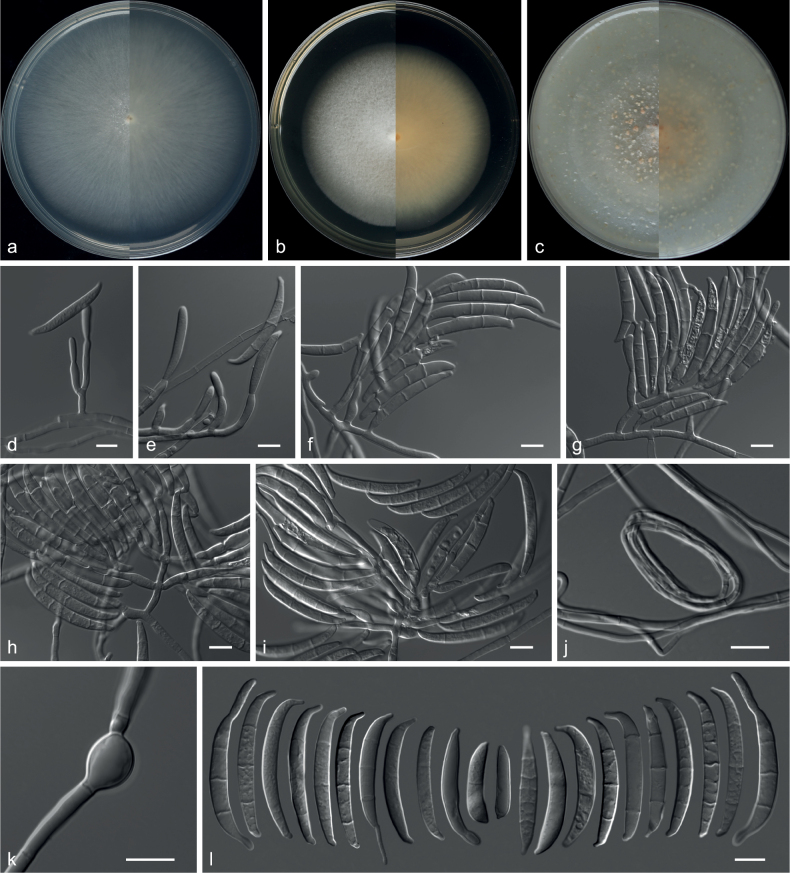
*Fusariumbrachypodum* (from ex-holotype CGMCC 3.20776) **a–c** upper and reverse views of cultures on PDA, MEA and OA 14 d after inoculation **d, e** conidiophores and phialides on aerial mycelium **f–i** sporodochia, sporodochial conidiophores and conidia **j** spiral hyphae **k** chlamydospores **l** conidia. Scale bars: 10 µm (**d–l**).

### ﻿Niessliaceae Kirschst.

***Niesslia* Auersw.**

The genus *Niesslia* was established in 1869, with the type species *N.chaetomium* (Auerswald 1869). *Niesslia* is characterised by tuberculate perithecia, surrounded by brown, septate setae, clavate asci and filiform ascospores (Auerswald 1869). This genus is one of the more species-rich genera of ascomycetes, but has received relatively little taxonomic attention. Members of the genus are mostly saprophytic and globally distributed (Gams et al. 2019).

#### ﻿ Niesslia guizhouensis

Taxon classificationFungiHypocrealesNiessliaceae

Zhi.Y. Zhang, Y.F. Han & Z.Q. Liang sp. nov.

4DFB2672-2AC5-5F7A-B876-AFA8060EE5DC

: 844174

Fig. 26

##### Etymology.

In reference to Guizhou, the Province where the type specimen was obtained.

##### Type.

China: Guizhou Province, Guiyang City, North Campus of Guizhou University 26°44'37"N, 106°67'46"E, soil, 13 Sep 2019, Z.Y. Zhang (HMAS 351877 holotype designated here, ex-type living culture CGMCC 3.20780 = GZUIFR 21.912); ibid., GZUIFR 21.913.

##### Description.

***Culture characteristics*** (14 d at 25 °C): ***Colony on PDA*** 26–27 mm diam., white (1A1), texture velvety, nearly round, margin entire; reverse white (1A1). ***Colony on MEA*** 14–16 mm diam., orange white (5A2), aerial mycelia sparse, compact, rugged, cracked, margin undulated; reverse brownish-yellow (5C8) to white (5A1) from centre to margin. ***Colony on OA*** 36 mm diam., white (1A1), with a light-coloured margin, felty, compact, plicated, convex, margin entire to undulate; reverse white (1A1).

***Hyphae*** branched, septate, hyaline, smooth, 0.5–2.0 μm diam. ***Sporulation*** abundant, nematogenous to synnematogenous. ***Phialides*** from hyphae, hyphal coils, fertile, acerose at the moderately thick-walled base, sometimes bending, hardly widening above and tapering to 0.5–1 μm at the tip, 10.5–79.0 × 1.0–3.0 µm. ***Conidia*** adhering to slimy heads, cylindrical, smooth- and thin-walled, 3.0–7.5 × 1.0–2.5 μm (av. 5.3 × 1.9 μm, n = 50). ***Chlamydospores*** not observed. ***Sexual morph*** unknown.

##### Additional specimens examined.

China: Guizhou Province, Guiyang City, Qianlingshan Park 26°59'03"N, 106°69'57"E, soil, 13 Sep 2019, Z.Y. Zhang, GZUIFR 21.914.

##### Notes.

**Figure 25.**
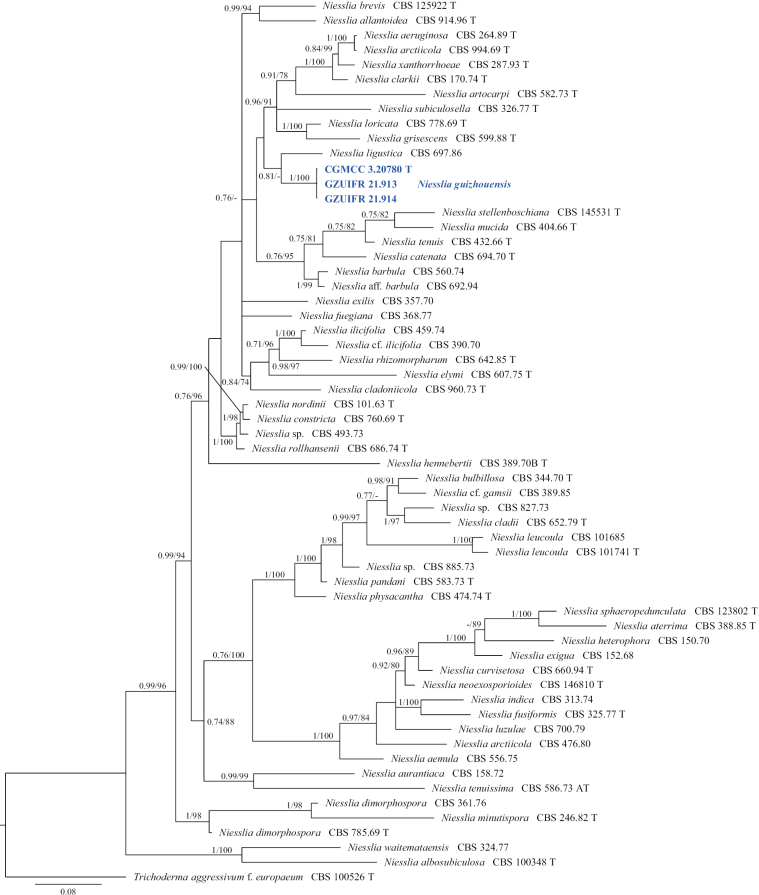
Concatenated phylogeny of the ITS, LSU, *EF1A* and *ACT* gene regions of species in *Niesslia*. Sixty strains are used. The tree is rooted in Trichodermaaggressivumf.europaeum (CBS 100526). The tree topology of the BI was similar to the ML analysis. Bayesian posterior probability (≥ 0.7) and ML bootstrap values (≥ 70%) and are indicated along branches (PP/ML). Novel species are in blue and bold font and “T” indicates type derived sequences.

*Niessliaguizhouensis* is phylogenetically related to *N.ligustica*, as demonstrated in Fig. 25, but can be differentiated from it by the absence of pigmented cells (Gams et al. 2019). In addition, the colony of *N.guizhouensis* on MEA are white with a white reverse, whereas those of *N.ligustica* are white or sometimes pale orange with a pale luteous to ochraceous reverse (Gams et al. in 2019).

**Figure 26.**
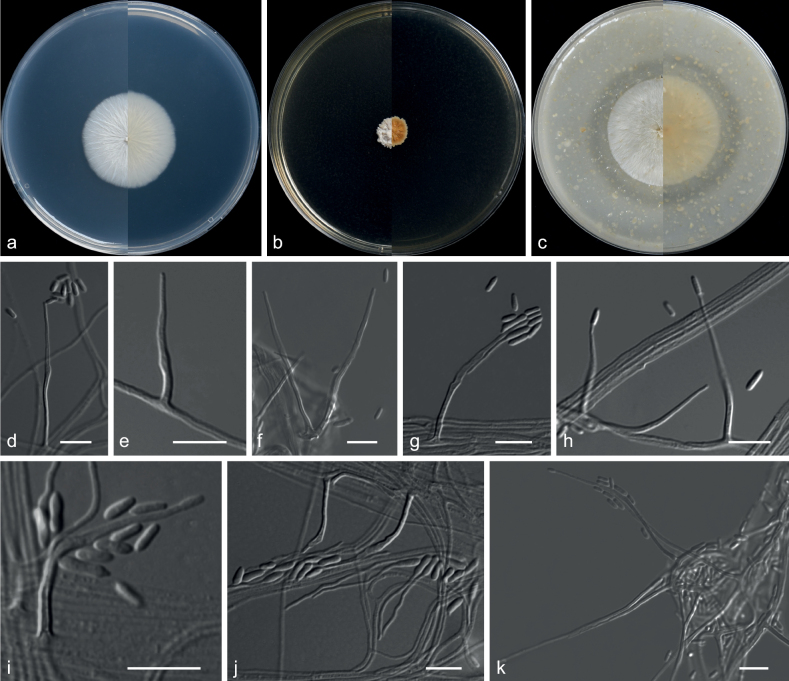
*Niessliaguizhouensis* (from ex-holotype CGMCC 3.20780) **a–c** upper and reverse views of cultures on PDA, MEA and OA 14 d after inoculation **d–j** monocillium-like conidiophores and conidia **k** hyphal coil with conidiophores. Scale bars: 10 µm (**d–k**).

### ﻿Xylariales Nannf.

**Microdochiaceae Hern.-Restr., Crous & J.Z. Groenew.**

***Idriella* P.E. Nelson & S. Wilh.**

*Idriella* comprises soil-inhabiting hyphomycetes and terrestrial species worldwide (Hyde et al. 2020b). The genus is characterised by brown, aseptate conidiophores and polyblastic conidiogenous cells with hyaline, unicellular, smooth, lunate, curved conidia in the heads (Hernández-Restrepo et al. 2016). Although the genus *Idriella* includes 30 species, molecular data are available for only four species and, based on the results of phylogenetic analyses, three of these species have been moved out as type species and new genera have been established (Castañeda-Ruiz and Kendrick 1991; Hernández-Restrepo et al. 2016). The taxonomic status of species morphologically similar to these three species is debatable (Castañeda-Ruiz and Kendrick 1991; Hernández-Restrepo et al. 2016).

#### ﻿ Idriella chlamydospora

Taxon classificationFungiXylarialesMicrodochiaceae

Zhi.Y. Zhang, Y.F. Han & Z.Q. Liang sp. nov.

E8A2725A-69F9-52B5-A363-6D500791FF11

: 844175

Fig. 28

##### Etymology.

Refers to the species that only produces chlamydospores.

##### Type.

China: Guangdong Province, Guangzhou City, Nanfang Hospital of Southern Medical University 23°19'14"N, 113°32'93"E, soil, 24 Aug 2019, Z.Y. Zhang (HMAS 351879 holotype designated here, ex-type living culture CGMCC 3.20778 = GZUIFR 21.921).

##### Description.

***Culture characteristics*** (14 d at 25 °C): ***Colony on PDA*** 40–41 mm diam., grey (30F1–30E1), flat, felty to pulverulent, nearly round, margin entire; reverse grey (29F1). ***Colony on MEA*** 31–33 mm diam., grey (6F1), compact, plicated, nearly round, margin entire; reverse soot brown (5F5) from centre to margin. ***Colony on OA*** 38 mm diam., grey (30F1) with a white circle, plicated, nearly round, margin entire; reverse greenish-grey (30E2).

***Hyphae*** branched, septate, hyaline, smooth, 1.0–3.0 μm diam. ***Chlamydospores*** arising in axenic culture on PDA, MEA and OA, moniliform, 1–2-septate, brown, 7.5–20.0 × 6.5–11.0 µm (av. 18.5 × 8.6 μm, n = 50). ***Conidia*** were not observed. ***Sexual morph*** unknown.

##### Additional specimens examined.

China: Guangdong Province, Guangzhou City, South Campus of Sun Yat-sen University 23°10'04"N, 113°29'95"E, soil, 24 Aug 2019, Z.Y. Zhang, GZUIFR 21.922.

##### Notes.

**Figure 27.**
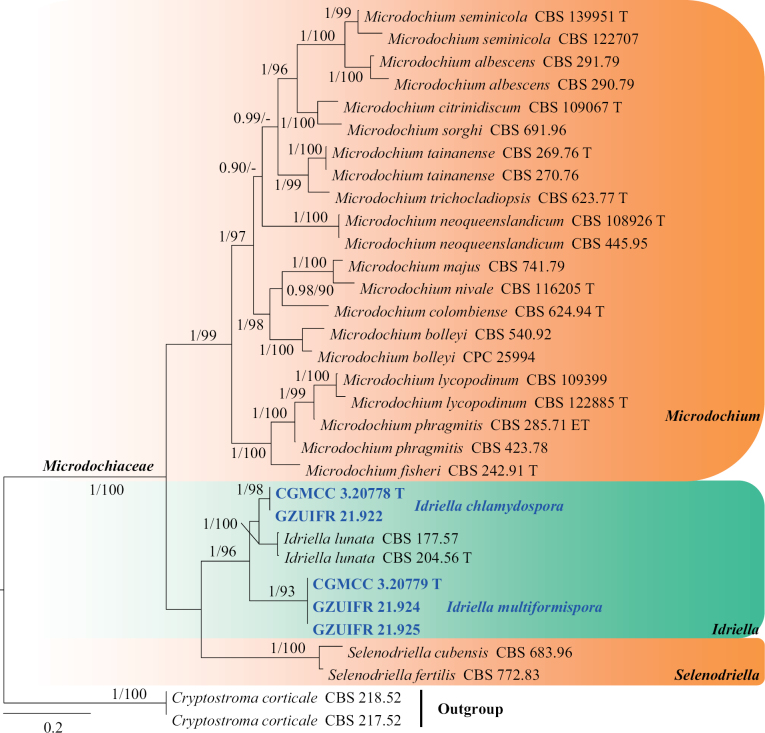
Concatenated phylogeny of the ITS, LSU, *TUB* and *RPB2* gene regions of species in Microdochiaceae. Thirty-two strains are used. The tree is rooted in *Cryptostromacorticale* (CBS 218.52 and CBS 217.52). The tree topology of the BI was similar to the ML analysis. Bayesian posterior probability (≥ 0.8) and ML bootstrap values (≥ 80%) are indicated along branches (PP/ML). Novel species are in blue and bold font, and “T” indicates type derived sequences.

*Idriellachlamydospora* was isolated from soil in China. Phylogenetically, the new isolates CGMCC 3.20778 and GZUIFR 21.922 formed a single clade with a strongly-supported value and were nested in the genus *Idriella* (Fig. 27). However, morphologically, *I.chlamydospora* differs from other species in the genus *Idriella* in that it only produces chlamydospores.

**Figure 28.**
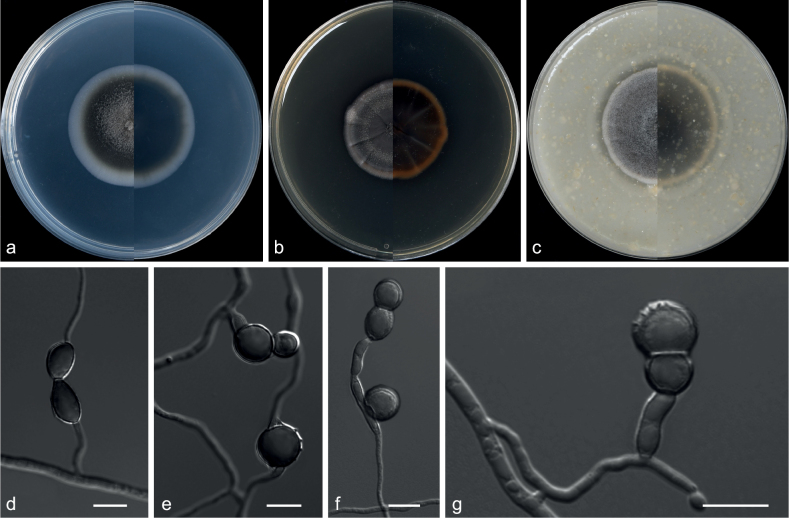
*Idriellachlamydospora* (from ex-holotype CGMCC 3.20778) **a–c** upper and reverse views of cultures on PDA, MEA and OA 14 d after inoculation **d–g** chlamydospores. Scale bars: 10 µm (**d–g**).

#### ﻿ Idriella multiformispora

Taxon classificationFungiXylarialesMicrodochiaceae

Zhi.Y. Zhang, Y.F. Han & Z.Q. Liang sp. nov.

E667E26F-FDD5-5A71-8B8B-0C5497690C17

: 844176

Fig. 29

##### Etymology.

Referring to the multiform conidia.

##### Type.

China: Jiangxi Province, Nanchang City, Nanchang People’s Park 28°68'12"N, 115°91'35"E, soil, 13 Aug 2019, Z.Y. Zhang (HMAS 351880 holotype designated here, ex-type living culture CGMCC 3.20779 = GZUIFR 21.923).

##### Description.

***Culture characteristics*** (14 d at 25 °C): ***Colony on PDA*** 51 mm diam., grey (30F1) to dark green (30F4), felty, compact, margin entire to undulated; reverse dark green (30F4). ***Colony on MEA*** 27–30 mm diam., greenish-grey (30E2), flat, stellate striate with grey, margin entire to undulated; reverse dark green (30F4). ***Colony on OA*** 33–38 mm diam., greenish-grey (30E2), aerial mycelia dense, plicated, sectorisation, nearly round; reverse greenish-grey (30E2).

**Figure 29.**
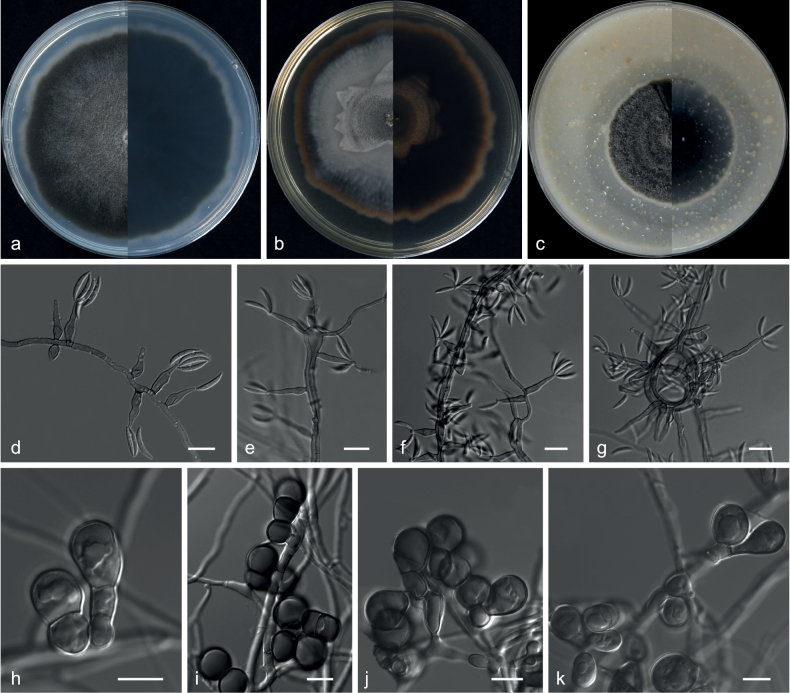
*Idriellamultiformispora* (from ex-holotype CGMCC 3.20779) **a–c** upper and reverse views of cultures on PDA, MEA and OA 14 d after inoculation **d–f** conidiogenous cells and conidia **g** hyphal coil with phialides **h–k** chlamydospores. Scale bars: 10 µm (**d–k**).

***Hyphae*** branched, septate, hyaline, smooth, 1.0–4.0 μm diam. ***Conidiophores*** reduced to conidiogenous cells. ***Conidiogenous cells*** numerous, borne on hyphae or hyphal coil, erect, straight or flexuous, lageniform, 9.5–25.5 µm long, 1.0–3.0 µm wide at the base, apex inflated or globose and 1.0–2.5 µm diam. ***Conidia*** lunate, sometimes acerose, pointed at each end, non-septate, smooth-walled, colourless, 8.5–13.5 × 1.0–2.0 µm (av. 11.6 × 1.7 μm, n = 50). ***Chlamydospores*** are borne on hyphae, moniliform or branched, 1–2-septate, brown, 12.5–22.5 × 6.5–11.5 µm (av. 21.4 × 10.5 μm, n = 50). ***Sexual morph*** unknown.

##### Additional specimens examined.

China: Jiangxi Province, Nanchang City, Qianhu Campus of Nanchang University 28°65'68"N, 115°80'12"E, soil, 13 Aug 2019, Z.Y. Zhang, GZUIFR 21.924, ibid., GZUIFR 21.925.

##### Notes.

According to Castañeda-Ruiz and Kendrick (1991), *Idriellamultiformispora* and *I.acerosa* share similarities in terms of their lunate conidia and moniliform or branched chlamydospores. While introducing *I.acerosa*, Castañeda-Ruiz and Kendrick (1991) also noted that it bears a resemblance to *I.desertorum*. However, molecular data on *I.acerosa* are not available. Later, Hernández-Restrepo et al. (2016) established the genus *Neoidriella*, based on the molecular analysis of *I.desertorum* and removed it from the genus *Idriella* as the type species. In this study, *I.multiformispora* was phylogenetically categorised within the genus *Idriella* (Fig. 27). Morphologically, *I.multiformispora* can be differentiated from *I.acerosa* by its fewer septate chlamydospores (Castañeda-Ruiz and Kendrick in 1991).

## Supplementary Material

XML Treatment for Echinocatena sinensis

XML Treatment for Aspergillus cylindricus

XML Treatment for Aspergillus doliiformis

XML Treatment for Penicillium fujianense

XML Treatment for Talaromyces guiyangensis

XML Treatment for Talaromyces jiangxiensis

XML Treatment for Talaromyces paecilomycetoides

XML Treatment for Nannizzia sinensis

XML Treatment for Pseudogymnoascus botryoides

XML Treatment for Pseudogymnoascus camphorae

XML Treatment for Pseudogymnoascus papyriferae

XML Treatment for Pseudogymnoascus zongqii

XML Treatment for Clonostachys shanghaiensis

XML Treatment for Cyanonectria bispora

XML Treatment for Fusarium brachypodum

XML Treatment for Niesslia guizhouensis

XML Treatment for Idriella chlamydospora

XML Treatment for Idriella multiformispora

## ﻿Additional information

### Author ORCIDs

Zhi-Yuan Zhang https://orcid.org/0000-0003-2031-7518

Xin Li https://orcid.org/0000-0001-7910-1469

Wan-Hao Chen https://orcid.org/0000-0001-7240-6841

Jian-Dong Liang https://orcid.org/0000-0002-3939-3900

Yan-Feng Han https://orcid.org/0000-0002-8646-3975

### Data availability

All of the data that support the findings of this study are available in the main text or Supplementary Information.

## Supplementary materials

Supplementary material 1 Strain numbers and sequence accession numbers of new isolates Data type table (word document) https://binary.pensoft.net/file/870738This dataset is made available under the Open Database License (http://opendatacommons.org/licenses/odbl/1.0/). The Open Database License (ODbL) is a license agreement intended to allow users to freely share, modify, and use this Dataset while maintaining this same freedom for others, provided that the original source and author(s) are credited.Zhi-Yuan Zhang, Xin Li, Wan-Hao Chen, Jian-Dong Liang, Yan-Feng Han

Supplementary material 2 Strains used in this study Data type table (excel document) https://binary.pensoft.net/file/870739This dataset is made available under the Open Database License (http://opendatacommons.org/licenses/odbl/1.0/). The Open Database License (ODbL) is a license agreement intended to allow users to freely share, modify, and use this Dataset while maintaining this same freedom for others, provided that the original source and author(s) are credited.Zhi-Yuan Zhang, Xin Li, Wan-Hao Chen, Jian-Dong Liang, Yan-Feng Han

Supplementary material 3 The concatenated sequences dataset Data type .zip file https://binary.pensoft.net/file/870740This dataset is made available under the Open Database License (http://opendatacommons.org/licenses/odbl/1.0/). The Open Database License (ODbL) is a license agreement intended to allow users to freely share, modify, and use this Dataset while maintaining this same freedom for others, provided that the original source and author(s) are credited.Zhi-Yuan Zhang, Xin Li, Wan-Hao Chen, Jian-Dong Liang, Yan-Feng Han
