# Supplementary material for: ﻿Culturable fungi from urban soils in China II, with the description of 18 novel species in Ascomycota (Dothideomycetes, Eurotiomycetes, Leotiomycetes and Sordariomycetes)
Source: MycoKeys. 2023 Jun 29;98:167–220. doi: 10.3897/mycokeys.98.102816 (PMC10326621; doi:10.3897/mycokeys.98.102816)
Supplement: Supplementary material 1 — Strain numbers and sequence accession numbers of new isolates [file mycokeys-98-167-s001.doc]

Table S1 Strain numbers and sequence accession numbers of new isolates.

| Species name | Strain number | ITS | LSU | *TUB* | *RPB2* | *EF1A* | *CaM* | *TSR1* | *TEF3* | *RP 60S L1* | *MCM7* | *ACT* |
| --- | --- | --- | --- | --- | --- | --- | --- | --- | --- | --- | --- | --- |
| *Echinocatena sinensis* | CGMCC 3.20775 = GZUIFR 21.900^T^ | OL897006 | OL897048 | ON569026 | ON568948 | ON568898 | – | – | – | – | – | – |
|  | GZUIFR 21.901 | OL897007 | OL897049 | ON569027 | ON568949 | ON568899 | – | – | – | – | – | – |
|  | GZUIFR 21.902 | OL897008 | OL897050 | ON569028 | ON568950 | ON568900 | – | – | – | – | – | – |
|  | GZUIFR 21.903 | OL897009 | OL897051 | ON569029 | ON568951 | ON568901 | – | – | – | – | – | – |
| *Aspergillus cylindricus* | CGMCC 3.20771 = GZUIFR 21.887^T^ | OL896995 | OL897037 | ON569030 | ON568952 | – | ON568876 | ON569008 | – | – | – | – |
|  | GZUIFR 21.888 | OL896996 | OL897038 | ON569031 | ON568953 | – | ON568877 | ON569009 | – | – | – | – |
|  | GZUIFR 21.889 | OL896997 | OL897039 | ON569032 | ON568954 | – | ON568878 | ON569010 | – | – | – | – |
| *Aspergillus doliiformis* | CGMCC 3.20772 = GZUIFR 21.885^T^ | OL896998 | OL897040 | ON569033 | ON568955 | – | ON568879 | ON569011 | – | – | – | – |
|  | GZUIFR 21.883 | OL896999 | OL897041 | ON569034 | ON568956 | – | ON568880 | ON569012 | – | – | – | – |
|  | GZUIFR 21.884 | OL897000 | OL897042 | ON569035 | ON568957 | – | ON568881 | ON569013 | – | – | – | – |
|  | GZUIFR 21.886 | OL897001 | OL897043 | ON569036 | ON568958 | – | ON568882 | ON569014 | – | – | – | – |
| *Penicillium fujianense* | CGMCC 3.20781 = GZUIFR 21.880^T^ | OL897024 | OL897066 | ON569037 | – | – | ON568883 | ON569015 | – | – | – | – |
|  | GZUIFR 21.881 | OL897025 | OL897067 | ON569038 | – | – | ON568884 | ON569016 | – | – | – | – |
|  | GZUIFR 21.882 | OL897026 | OL897068 | ON569039 | – | – | ON568885 | ON569017 | – | – | – | – |
| *Talaromyces guiyangensis* | CGMCC 3.20782 = GZUIFR 21.890^T^ | OL897027 | OL897069 | ON569040 | ON568959 | – | ON568886 | ON569018 | – | – | – | – |
|  | GZUIFR 21.891 | OL897028 | OL897070 | ON569041 | ON568960 | – | ON568887 | ON569019 | – | – | – | – |
| *Talaromyces jiangxiensis* | CGMCC 3.20783 = GZUIFR 21.892^T^ | OL897029 | OL897071 | ON569042 | ON568961 | – | ON568888 | ON569020 | – | – | – | – |
|  | GZUIFR 21.893 | OL897030 | OL897072 | ON569043 | ON568962 | – | ON568889 | ON569021 | – | – | – | – |
| *Talaromyces paecilomycetoides* | CGMCC 3.20785 = GZUIFR 21.894^T^ | OL897033 | OL897075 | ON569044 | ON568963 | – | ON568890 | ON569022 | – | – | – | – |
|  | GZUIFR 21.895 | OL897034 | OL897076 | ON569045 | ON568964 | – | ON568891 | ON569023 | – | – | – | – |
|  | GZUIFR 21.896 | OL897035 | OL897077 | ON569046 | ON568965 | – | ON568892 | ON569024 | – | – | – | – |
|  | GZUIFR 21.897 | OL897036 | OL897078 | ON569047 | ON568966 | – | ON568893 | ON569025 | – | – | – | – |
| *Arthroderma quadrifidum/redellii* | CGMCC 3.20862 | ON365893 | ON366320 | ON569048 | – | – | – | – | ON568991 | ON568931 | – | – |
|  | GZUIFR 22.052 | ON365894 | ON366321 | ON569049 | – | – | – | – | ON568992 | ON568932 | – | – |
|  | CGMCC 3.20863 | ON365895 | ON366322 | ON569050 | – | – | – | – | ON568993 | ON568933 | – | – |
|  | GZUIFR 22.050 | ON365896 | ON366323 | ON569051 | – | – | – | – | ON568994 | ON568934 | – | – |
|  | CGMCC 3.20859 | ON365897 | ON366324 | ON569052 | – | – | – | – | ON568995 | ON568935 | – | – |
|  | GZUIFR 22.051 | ON365898 | ON366325 | ON569053 | – | – | – | – | ON568996 | ON568936 | – | – |
|  | CGMCC 3.20861 | ON365899 | ON366326 | ON569054 | – | – | – | – | ON568997 | ON568937 | – | – |
|  | GZUIFR 22.053 | ON365900 | ON366327 | ON569055 | – | – | – | – | ON568998 | ON568938 | – | – |
| *Arthroderma gertleri* | GZUIFR 22.059 | ON365901 | ON366328 | ON569056 | – | – | – | – | ON568999 | ON568939 | – | – |
| *Arthroderma uncinatum* | CGMCC 3.20865 | ON365902 | ON366329 | ON569057 | – | – | – | – | ON569000 | ON568940 | – | – |
| *Arthroderma ciferrii* | CGMCC 3.20860 | ON365903 | ON366330 | ON569058 | – | – | – | – | ON569001 | ON568941 | – | – |
| *Arthroderma thuringiensis* | CGMCC 3.20864 | ON365904 | ON366331 | ON569059 | – | – | – | – | ON569002 | ON568942 | – | – |
| *Arthroderma tuberculatum* | GZUIFR 22.060 | ON365905 | ON366332 | ON569060 | – | – | – | – | ON569003 | ON568943 | – | – |
| *Nannizzia sinensis* | CGMCC 3.20873 = GZUIFR 22.012^T^ | ON365906 | ON366333 | ON569061 | – | – | – | – | ON569004 | ON568944 | – | – |
|  | GZUIFR 22.054 | ON365907 | ON366334 | ON569062 | – | – | – | – | ON569005 | ON568945 | – | – |
|  | GZUIFR 22.055 | ON365908 | ON366335 | ON569063 | – | – | – | – | ON569006 | ON568946 | – | – |
|  | GZUIFR 22.056 | ON365909 | ON366336 | ON569064 | – | – | – | – | ON569007 | ON568947 | – | – |
| *Pseudogymnoascus botryoides* | CGMCC 3.20875 = GZUIFR 22.024^T^ | ON365910 | ON366337 | – | ON568967 | ON568902 | – | – | – | – | – | – |
|  | GZUIFR 22.044 | ON365911 | ON366338 | – | ON568968 | ON568903 | – | – | – | – | – | – |
|  | GZUIFR 22.045 | ON365912 | ON366339 | – | ON568969 | ON568904 | – | – | – | – | – | – |
|  | GZUIFR 22.046 | ON365913 | ON366340 | – | ON568970 | ON568905 | – | – | – | – | – | – |
| *Pseudogymnoascus camphorae* | CGMCC 3.20876 = GZUIFR 22.021^T^ | ON365914 | ON366341 | – | ON568971 | ON568906 | – | – | – | – | ON568921 | – |
|  | GZUIFR 22.049 | ON365915 | ON366342 | – | ON568972 | ON568907 | – | – | – | – | ON568922 | – |
| *Pseudogymnoascus papyriferae* | CGMCC 3.20877 = GZUIFR 22.020^T^ | ON365916 | ON366343 | – | ON568973 | ON568908 | – | – | – | – | ON568923 | – |
| *Pseudogymnoascus yunnanensis* | CGMCC 3.20879 | ON365917 | ON366344 | – | ON568974 | ON568909 | – | – | – | – | ON568924 | – |
|  | GZUIFR 22.047 | ON365918 | ON366345 | – | ON568975 | ON568910 | – | – | – | – | ON568925 | – |
|  | CGMCC 3.20880 | ON365922 | ON366349 | – | ON568979 | ON568914 | – | – | – | – | ON568929 | – |
|  | GZUIFR 22.048 | ON365923 | ON366350 | – | ON568980 | ON568915 | – | – | – | – | ON568930 | – |
| *Pseudogymnoascus zongqii* | CGMCC 3.20878 = GZUIFR 22.025^T^ | ON365919 | ON366346 | – | ON568976 | ON568911 | – | – | – | – | ON568926 | – |
|  | GZUIFR 22.042 | ON365920 | ON366347 | – | ON568977 | ON568912 | – | – | – | – | ON568927 | – |
|  | GZUIFR 22.043 | ON365921 | ON366348 | – | ON568978 | ON568913 | – | – | – | – | ON568928 | – |
| *Clonostachys shanghaiensis* | CGMCC 3.20773 = GZUIFR 21.915^T^ | OL897002 | OL897044 | – | – | – | – | – | – | – | – | – |
|  | GZUIFR 21.916 | OL897003 | OL897045 | – | – | – | – | – | – | – | – | – |
| *Cyanonectria bispora* | CGMCC 3.20774 = GZUIFR 21.908^T^ | OL897004 | OL897046 | ON569065 | ON568981 | – | ON568894 | – | – | – | – | – |
|  | GZUIFR 21.909 | OL897005 | OL897047 | ON569066 | ON568982 | – | ON568895 | – | – | – | – | – |
| *Fusarium brachypodum* | CGMCC 3.20776 = GZUIFR 21.910^T^ | OL897010 | OL897052 | ON569067 | ON568983 | ON568916 | ON568896 | – | – | – | – | – |
|  | GZUIFR 21.911 | OL897011 | OL897053 | ON569068 | ON568984 | ON568917 | ON568897 | – | – | – | – | – |
| *Niesslia guizhouensis* | CGMCC 3.20780 = GZUIFR 21.912^T^ | OL897021 | OL897063 | – | ON568985 | ON568918 | – | – | – | – | – | ON568873 |
|  | GZUIFR 21.913 | OL897022 | OL897064 | – | ON568986 | ON568919 | – | – | – | – | – | ON568874 |
|  | GZUIFR 21.914 | OL897023 | OL897065 | – | ON568987 | ON568920 | – | – | – | – | – | ON568875 |
| *Idriella chlamydospora* | CGMCC 3.20778 = GZUIFR 21.921^T^ | OL897016 | OL897058 | ON569069 | – | – | – | – | – | – | – | – |
|  | GZUIFR 21.922 | OL897017 | OL897059 | ON569070 | – | – | – | – | – | – | – | – |
| *Idriella multiformispora* | CGMCC 3.20779 = GZUIFR 21.923^T^ | OL897018 | OL897060 | ON569071 | ON568988 | – | – | – | – | – | – | – |
|  | GZUIFR 21.924 | OL897019 | OL897061 | ON569072 | ON568989 | – | – | – | – | – | – | – |
|  | GZUIFR 21.925 | OL897020 | OL897062 | ON569073 | ON568990 | – | – | – | – | – | – | – |

^T^ Ex-type specimen.
